# Multidimensional sleep health: risk of cardiovascular disease and mortality, and mediation by plasma proteins

**DOI:** 10.7189/jogh.16.04349

**Published:** 2026-10-23

**Authors:** Zichun Wei, Qi Xiao, Siyu Wang, Luxuan Liu, Deguo Meng, Zhi Mao, Ruogu Lu, Rongyao Hou, Xiaoyan Zhu

**Affiliations:** 1 Department of Critical Care Medicine, The Affiliated Hospital of Qingdao University, Qingdao, China; 2 Department of Neurology, The Affiliated Hospital of Qingdao University, Qingdao, China; 3 Department of Neuroscience Critical Care Unit, Johns Hopkins Hospital, Baltimore, USA; 4 Department of Critical Care Medicine, The First Medical Center of PLA General Hospital, Beijing, China; 5 Medical Innovation Research Department, Chinese PLA General Hospital, Beijing, China; 6 Department of Neurology, The Affiliated Hiser Hospital of Qingdao University, Qingdao, China

**Keywords:** sleep health, cardiovascular disease, mortality, plasma proteomics, mediation analysis

## Abstract

**Background:**

Sleep health is a recognised determinant of cardiovascular health, but most previous research has examined individual sleep traits in relation to cardiovascular disease and mortality. The molecular pathways linking multidimensional sleep health to these outcomes remain insufficiently explored. We aimed to examine these associations and identify circulating proteins showing potential mediation signals in these relationships.

**Methods:**

We analysed data on 257,509 individuals from the UK Biobank, including 26,984 with available plasma proteomic data. Using baseline questionnaire data collected by UK Biobank, we calculated a seven-component sleep health score (SHS) and derived data-driven sleep patterns using K-prototypes clustering. We used multivariable Cox proportional hazards models to examine the associations of the SHS and, separately, the sleep patterns with incident coronary heart disease, stroke, atrial fibrillation and heart failure, as well as cardiovascular and overall mortality. Lastly, we identified SHS-related proteins using linear regression, with representative proteins selected using least absolute shrinkage and selection operator regression, and mediation analyses performed.

**Results:**

Higher SHS was associated with lower risks of coronary heart disease, stroke, atrial fibrillation, heart failure, cardiovascular mortality, and overall mortality, with the strongest associations observed for heart failure and coronary heart disease. Mixed sleep disturbance and non-restorative sleep patterns were associated with higher risks across multiple outcomes. Proteomic analyses identified 188 SHS-related proteins, mainly enriched in inflammatory, immune, and chemotactic pathways. Exploratory mediation analyses identified 34 proteins with potential mediation signals, among which HGF, IGF2R, and FABP4 showed recurrent cross-outcome signals.

**Conclusions:**

Poor multidimensional sleep health was associated with higher cardiovascular disease and mortality risks, with exploratory proteomic analyses suggesting potential involvement of circulating proteins related to inflammation, immunity, chemotaxis, and metabolic dysregulation. These findings support multidimensional sleep assessment as a population-level prevention strategy and provide candidate molecular pathways for future mechanistic and interventional studies.

Cardiovascular disease (CVD) is the leading cause of the global disease burden and premature mortality [1], which makes exploring their risk factors crucial for global health. Previous studies have linked sleep characteristics, including abnormal sleep duration [2], insomnia symptoms [3], excessive daytime sleepiness [4,5], and abnormal chronotype preference [6], with adverse cardiovascular events and mortality risk. Recognising this, the American Heart Association has recently formally incorporated sleep into their guidance for assessing cardiovascular health [7]. Yet prior research into these associations has primarily focused on individual factors such as sleep duration or insomnia, thereby overlooking the coexistence and clustering of multiple sleep problems in real-world populations [8,9] and the multidimensional nature of sleep health [10].

Buysse conceptualised sleep health across five dimensions – satisfaction, alertness, timing, efficiency, and duration, while subsequent studies have further highlighted the importance of rhythmicity [10–12]. A growing body of evidence suggests that sleep abnormalities often occur as clinically meaningful composite phenotypes, rather than in isolation. For instance, insomnia with short sleep duration has been regarded as a more severe biological phenotype [13], comorbid insomnia and sleep apnoea have been associated with higher health risks than either condition alone [14], and insomnia has been found to commonly coexists with circadian misalignment and other comorbid conditions [15,16]. Taken together, this would indicate that a multidimensional sleep health framework may better capture the cumulative burden of sleep disturbances and provide a more comprehensive approach for evaluating cardiovascular risk.

Beyond epidemiologic associations, the biological mechanisms through which sleep influences cardiovascular events and mortality remain poorly understood. As downstream functional products of gene expression, plasma proteins cannot only serve as clinical biomarkers, but also provide direct insight into disease pathophysiology and potential therapeutic targets [17,18]. Proteomic studies have primarily focused on identifying disease-related proteins, improving early risk prediction, or prioritising potential therapeutic targets, while large-scale plasma proteomic resources such as those from the UK Biobank have further demonstrated the utility of proteomics for disease characterisation and predictive modelling [18–21]. However, proteomic studies of upstream behavioural exposures, particularly multidimensional sleep health, remain limited. Given that sleep may influence cardiovascular health through inflammation, immunity, metabolism, and vascular homeostasis, identifying sleep-related protein signatures and potential mediating proteins may help clarify the molecular links between sleep health and cardiovascular outcomes [22,23].

We conducted this prospective analysis of UK Biobank data to examine the associations of multidimensional sleep health with cardiovascular events and mortality, identify circulating proteins associated with sleep health, explore potential protein-mediated pathways, and assess the predictive value of sleep-related proteins by integrating epidemiologic, proteomic, mediation, and machine learning (ML) approaches.

## METHODS

The UK Biobank is a large-scale prospective cohort study that recruited more than 500,000 participants aged 37–73 years in the UK between 2006 and 2010, with the aim of improving the prevention, diagnosis, and treatment of various diseases. At baseline (2006–2010), participants completed a detailed touchscreen questionnaire covering lifestyle and health-related factors, including sleep characteristics, underwent physical assessments, and provided biological samples. The UK Biobank collected the baseline data and linked participants’ records to hospital admission records, primary care data, and national death registries. For this secondary analysis, we required complete, valid sleep-exposure data for sleep-pattern clustering and complete sleep-exposure and covariate data for the primary association analyses. We excluded participants with missing or invalid sleep-exposure data and, for the primary association analyses, those with missing key covariates, including unknown ethnicity. Proteomic analyses were restricted to participants with available plasma proteomic measurements. We confirm that, as a secondary data analysis of the UK Biobank data, our study aligns with the Journal of Global Health’s GRABDROP guidelines [24] (Table S1 in the **Online Supplementary Document**).

### Definition of exposure

We assessed sleep health at baseline using a seven-component sleep health score (SHS) derived from a recent UK Biobank study of multidimensional sleep health [25]. The SHS is based on seven self-reported sleep-related factors available in the UK Biobank: sleep duration, insomnia symptoms, non-restorative sleep, chronotype, napping, snoring, and daytime dozing. These components map onto the major sleep health domains available in the UK Biobank questionnaire, including sleep duration, sleep continuity, sleep restoration, circadian timing, daytime alertness or impairment, sleep-disordered breathing symptoms, and daytime sleep behaviour (Table S2 in the **Online Supplementary Document**).

We categorised sleep duration as short (<7 hours/day), medium (7–8 hours/day), or long (>8 hours/day), chronotype as morning, intermediate, or evening type, and other sleep traits as binary variables. In the primary analysis, we grouped ‘sometimes’ responses for napping, insomnia symptoms, and daytime dozing with ‘never/rarely’ responses to focus on more persistent symptoms and to reduce potential misclassification from transient or occasional sleep complaints. The SHS assigned 1 point to each favourable characteristic: 7–8 hours of sleep, absence of frequent insomnia symptoms, no snoring, no habitual daytime dozing or napping, restorative sleep, and intermediate chronotype. All components were equally weighted to create a simple and non-outcome-driven measure of multidimensional sleep health. The total SHS ranged from 0 to 7, with higher scores indicating healthier sleep. Participants were classified as having optimal (6–7), mildly impaired (5), moderately impaired (3–4), or severely impaired (0–2) sleep health.

### Proteomics data

Baseline plasma samples in the UK Biobank were collected between 2007 and 2010 and stored long-term at −80°C or in liquid nitrogen. Blood samples from a subset of UK Biobank participants were transported to the Olink Analysis Service in Sweden, where plasma proteins were quantified using proximity extension assay combined with next-generation sequencing. The detailed procedures for sample preparation, experimental assays, data processing, and quality control have been described in previous publications [19]. For this study, we excluded proteins with missing values in more than 20% of samples, leaving 2,911 proteins for downstream analyses. We imputed the remaining missing protein values using the K-nearest neighbours method and standardised protein measurements before regression analysis.

### Outcome definition

Based on prior studies [26,27], we studied six clinical outcomes during follow-up: coronary heart disease (CHD), stroke, atrial fibrillation (AF), heart failure (HF), CVD mortality, and overall mortality. We used UK Biobank’s first occurrence and death registry data fields to define the study outcomes. The UK Biobank derived the first occurrence fields from linked hospital inpatient, primary care, and death registry records and self-reported medical history (Table S3 in the **Online Supplementary Document**). We calculated follow-up time from the date of baseline assessment to the earliest date of the corresponding outcome event, death, loss to follow-up, or end of follow-up, whichever came first. We excluded participants with a history of the respective condition at baseline from each outcome-specific analysis.

### Covariates

We included age, sex, ethnicity, Townsend deprivation index (TDI), body mass index (BMI), employment status, educational level, smoking status, alcohol consumption, physical activity, and family history of heart disease, and stroke as covariates (Table S4 in the **Online Supplementary Document**). We treated age, TDI, BMI, and physical activity (total minutes per week) as continuous variables, and the remaining covariates as categorical variables. Among the latter, we classified ethnicity white or non-white; employment status as employed, retired, or unemployed/unable to work; educational level as high, moderate, low, or other; smoking status and alcohol consumption as never, former, or current smoker/drinker.

### Statistical analysis

We summarised baseline characteristics as medians (interquartile ranges) or percentages and compared groups using the Kruskal–Wallis, Pearson’ chi-squared, or Fisher’s exact tests, as appropriate. Multivariable Cox proportional hazards models examined associations of SHS and sleep patterns with each outcome, adjusting for the covariates described above. We modelled SHS continuously per one-point increase and categorically, using scores of 6–7 as the reference. Restricted cubic splines with three knots examined the shape of the associations. We evaluated individual sleep components using separate adjusted Cox models and assessed their relative importance using XGBoost-derived SHapley Additive exPlanations values. We derived five sleep patterns using K-prototypes clustering, with cluster number selected using the elbow method and Calinski–Harabasz index.

We assessed differences between analytic samples using standardised mean differences and examined associations by sex and age (>60 *vs*. ≤60 years), including interaction tests. Sensitivity analyses included competing-risk analyses where applicable, exclusion of outcomes occurring within the first year, multiple imputation of missing covariates using chained equations, and three alternative SHS definitions.

In the proteomic subset, we used multivariable linear regression to identify SHS-associated proteins. Least absolute shrinkage and selection operator (LASSO) regression used 10-fold cross-validation and lambda.min; proteins selected in all 1,000 repetitions were retained. We performed Gene Ontology functional and Kyoto Encyclopedia of Genes and Genomes pathway enrichment analyses and used elastic net regression to assess selection robustness. After re-evaluating SHS–outcome associations within this subset, we assessed individual proteins for exploratory mediation using a difference-in-coefficients approach on the log-hazard-ratio scale, with bootstrap confidence intervals. We also examined SHS × protein interaction terms.

For machine-learning analyses, we first randomly split participants into training and test sets in an 8:2 ratio, stratified by outcome status. Protein screening, LASSO selection, light gradient boosting machine development, hyperparameter tuning, and sequential forward selection were conducted within the training set, with 10-fold cross-validation. We evaluated the final models in the held-out test set and compared protein, demographic, and combined models using the area under the receiver operating characteristic curve. SHAP values were used to interpret protein contributions.

Further details on the statistical analyses are provided in Methods S1 in the **Online Supplementary Document**. We performed all analyses using *R*, version 4.5.0 (R Core team, Vienna, Austria), with two-sided tests and a false discovery rate (FDR) or Bonferroni-adjusted *P*-value <0.05 considered statistically significant.

## RESULTS

The primary analysis covered 257,509 of the 501,936 participants of the UK Biobank study after the exclusion of individuals with missing or invalid sleep exposure data (n = 92,172) and with missing key covariates (n = 152,255). For outcome-specific analyses, we further excluded participants with the corresponding disease at baseline, resulting in different sample sizes across endpoints (Figure S1 in the **Online Supplementary Document**). Proteomic analyses were restricted to 26,984 participants with available plasma proteomic measurements.

The median age was of the 257,509 participants was 57 years, 53.69% were female, and 95.40% were white (**Table 1**). Baseline characteristics showed a clear gradient across SHS categories. Lower SHS was associated with male sex, non-white ethnicity, greater socioeconomic disadvantage, lower education, unemployment or inability to work, higher BMI, lower physical activity, current smoking, lower alcohol consumption, and a higher prevalence of family history of CVD. Individual sleep components also showed consistent gradients across SHS categories, supporting the construct validity of the composite score. We further compared participants included in the primary analytic cohort with those excluded because of missing or invalid sleep exposure data or missing key covariates (Table S5 in the **Online Supplementary Document**). Overall, most assessed characteristics showed standardised mean differences below 0.1, suggesting no marked imbalance for most variables. The available sleep-related characteristics, including sleep duration, non-restorative sleep, chronotype, napping, insomnia, snoring, and daytime dozing, all showed small between-group differences. We observed moderate imbalances for age, employment status, educational level, TDI, and body mass index. These variables were included as covariates in the multivariable models. These findings suggest that although excluded participants were not completely identical to included participants, there was no evidence of substantial imbalance in most measured characteristics, particularly in the available sleep-related profiles.

**Table 1:** Baseline characteristics by SHS categories*

		Sleep health score				
	**Overall (n = 257,509)**	**6–7 (n = 136,637)**	**5 (n = 70,363)**	**3–4 (n = 47,509)**	**0–2 (n = 3,000)**	***P*-value**
**Age in years, Mdn (IQR)**	57.00 (49.00, 62.00)	56.00 (48.00, 62.00)	57.00 (50.00, 63.00)	57.00 (50.00, 62.00)	57.00 (50.00, 63.00)	<0.001
**Female**	53.69	55.50	51.51	52.22	45.87	<0.001
**White**	95.40	96.14	95.03	94.08	91.20	<0.001
**Employment**						<0.001
Employed	65.05	66.69	65.08	61.40	46.83	
Retired	28.74	28.46	28.96	29.07	31.60	
Unemployed/unable to work	6.21	4.85	5.96	9.54	21.57	
**Education**						<0.001
High	52.55	54.46	51.72	48.82	44.37	
Moderate	17.87	18.19	17.70	17.37	15.20	
Low	16.42	16.20	16.39	17.09	16.80	
Other	13.15	11.14	14.19	16.72	23.63	
**TDI, Mdn (IQR)**	−2.31 (−3.73, 0.14)	−2.43 (−3.80, −0.14)	−2.26 (−3.70, 0.21)	−2.01 (−3.58, 0.76)	−1.08 (−3.02, 2.11)	<0.001
**BMI in kg/m^2^, Mdn (IQR)**	26.52 (23.89, 29.57)	25.99 (23.60, 28.80)	26.83 (24.25, 29.93)	27.59 (24.81, 31.04)	29.57 (26.16, 33.68)	<0.001
**Physical activity in min/week, Mdn (IQR)**	100.00 (60.00, 180.00)	105.00 (60.00, 180.00)	100.00 (55.00, 180.00)	90.00 (50.00, 170.00)	70.00 (30.00, 145.00)	<0.001
**Smoking**						<0.001
Never	55.19	58.00	53.60	50.31	42.07	
Current	9.70	8.23	10.17	12.66	18.97	
Former	35.11	33.77	36.23	37.03	38.97	
**Alcohol**						<0.001
Never	3.65	3.29	3.82	4.32	5.73	
Current	93.20	94.12	93.01	91.23	86.50	
Former	3.15	2.59	3.17	4.45	7.77	
**Family history of heart disease and stroke**	58.28	56.66	59.06	61.38	64.37	<0.001
**Sleep duration in hours**						<0.001
Short (<7)	23.15	6.37	31.17	57.12	61.47	
Medium (7–8)	69.68	90.74	59.17	28.47	9.23	
Long (>8)	7.17	2.88	9.66	14.41	29.30	
**Non-restorative sleep**	17.53	6.56	21.46	39.79	72.57	<0.001
**Chronotype**						<0.001
Morning	26.69	15.92	36.49	42.66	34.40	
Intermediate	64.48	81.81	52.81	34.83	18.63	
Evening	8.83	2.27	10.70	22.51	46.97	
**Napping**	4.94	0.96	4.74	13.58	53.63	<0.001
**Insomnia**	26.84	7.84	35.00	65.52	88.20	<0.001
**Snoring**	36.81	20.78	49.16	61.69	83.13	<0.001
**Daytime dozing**	2.51	0.23	1.62	7.42	49.63	<0.001

BMI – body mass index, IQR – interquartile range, Mdn – median, SHS – sleep health score, TDI – Townsend deprivation index

*Presented as n (%) unless specified otherwise.

### Associations of SHS with cardiovascular events and mortality

In continuous analyses, each one-point increase in SHS was linked to lower risks of CHD (hazard ratio (HR) = 0.92; 95% confidence interval (CI) = 0.91, 0.93), stroke (HR = 0.96; 95% CI = 0.94, 0.98), AF (HR = 0.97; 95% CI = 0.96, 0.99), HF (HR = 0.91; 95% CI = 0.89, 0.93), CVD mortality (HR = 0.96; 95% CI = 0.94, 0.99), and overall mortality (HR = 0.95; 95% CI = 0.94, 0.96), with all associations remaining significant after FDR correction. Categorical analyses further showed a clear dose–response relationship emerged: compared with participants with optimal sleep health (6–7 score), progressively lower SHS was associated with increasing risks across all outcomes, with the strongest associations observed for HF, followed by CHD and overall mortality. Participants with SHS of 0–2 had markedly elevated risks, including a 61% higher risk of HF and a 48% higher risk of CHD. In contrast, risk elevations for stroke, AF, CVD mortality, and overall mortality were not evident or were only marginal at an SHS of 5, but became clearly apparent among participants with SHS <5 (**Figure 1**).

**Figure 1:**
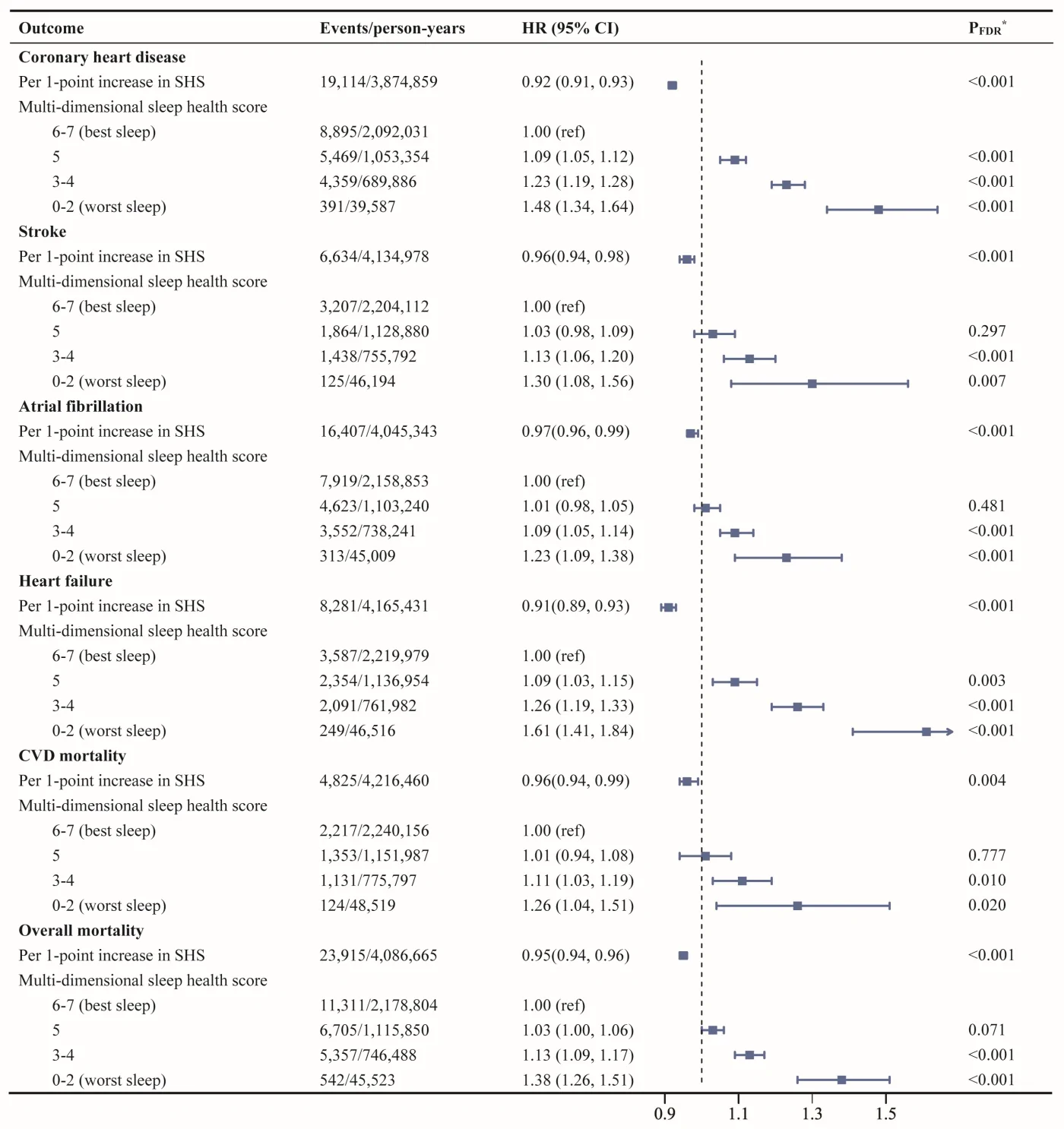
Associations of the SHS with risks of cardiovascular events and mortality. Blue squares represent estimated HRs and horizontal error bars indicate 95% CI. The vertical dashed line denotes the null value (HR = 1.0). Associations are shown both per one-point increase in the SHS and across sleep health score categories, with scores of 6–7 (best sleep) as the reference group. The HRs were estimated using multivariable Cox proportional hazards regression models adjusted for age, sex, ethnicity, TDI, BMI, employment status, educational level, smoking status, alcohol consumption, physical activity, and family history of heart disease and stroke. BMI – body mass index, CI – confidence interval, CVD – cardiovascular disease, FDR – false discovery rate, HR – hazard ratio, SHS – sleep health score, TDI – Townsend deprivation index. *P-values were based on two-sided tests and were adjusted for multiple comparisons using the FDR method.

These findings generally remained consistent in stratified and sensitivity analyses (Figures S3 and S4, and Tables S6–13 in the **Online Supplementary Document**). In detail, restricted cubic spline analyses showed broadly inverse and approximately linear associations between SHS and all outcomes, with steeper risk gradients at lower SHS levels. Analyses of individual sleep components suggested that the observed associations were not driven by a single sleep dimension, but reflected heterogeneous contributions from multiple sleep traits. The associations were largely consistent across sex and age groups, with no evidence of significant interaction after correction, and were essentially unchanged after applying competing-risk models or excluding events occurring within the first year of follow-up, after applying alternative SHS definitions, and after reclassifying ‘sometimes’ responses for insomnia symptoms, napping, and daytime dozing as unfavourable, coding morning and intermediate chronotypes as favourable, or excluding chronotype from the SHS. Multiple imputation analyses for missing covariates similarly yielded findings consistent with the complete-case analyses.

### Associations of sleep health patterns with cardiovascular events and mortality

We identified distinct sleep health patterns on the real-world co-occurrence structure of multidimensional sleep behaviours. Sleep disturbances were not distributed randomly across individuals, but instead aggregated into clinically interpretable profiles (**Figure 2**, Panel A). The obstructive symptom group (n = 85,046) was dominated by snoring (100.00%), suggesting a relatively specific obstructive-feature pattern. The mixed sleep disturbance group (n = 53,868) showed the most unfavourable and complex profile, with marked clustering of short sleep duration (73.19%) and insomnia (81.33%) along with a greater burden of additional adverse sleep traits, consistent with a multi-impairment phenotype. The morning chronotype group (n = 40,785) was characterised by predominant morning preference (91.03%) and otherwise comparatively favourable sleep features. The non-restorative sleep group (n = 22,146) was defined by universal non-restorative sleep (100.00%) and appeared to represent a distinct subjective sleep-quality phenotype. The remaining participants were assigned to the reference group (n = 55,664), which showed uniformly low prevalence across adverse sleep characteristics. Baseline characteristics differed significantly across these patterns, further supporting that the identified clusters captured meaningful heterogeneity in multidimensional sleep health (Table S14 in the **Online Supplementary Document**).

**Figure 2:**
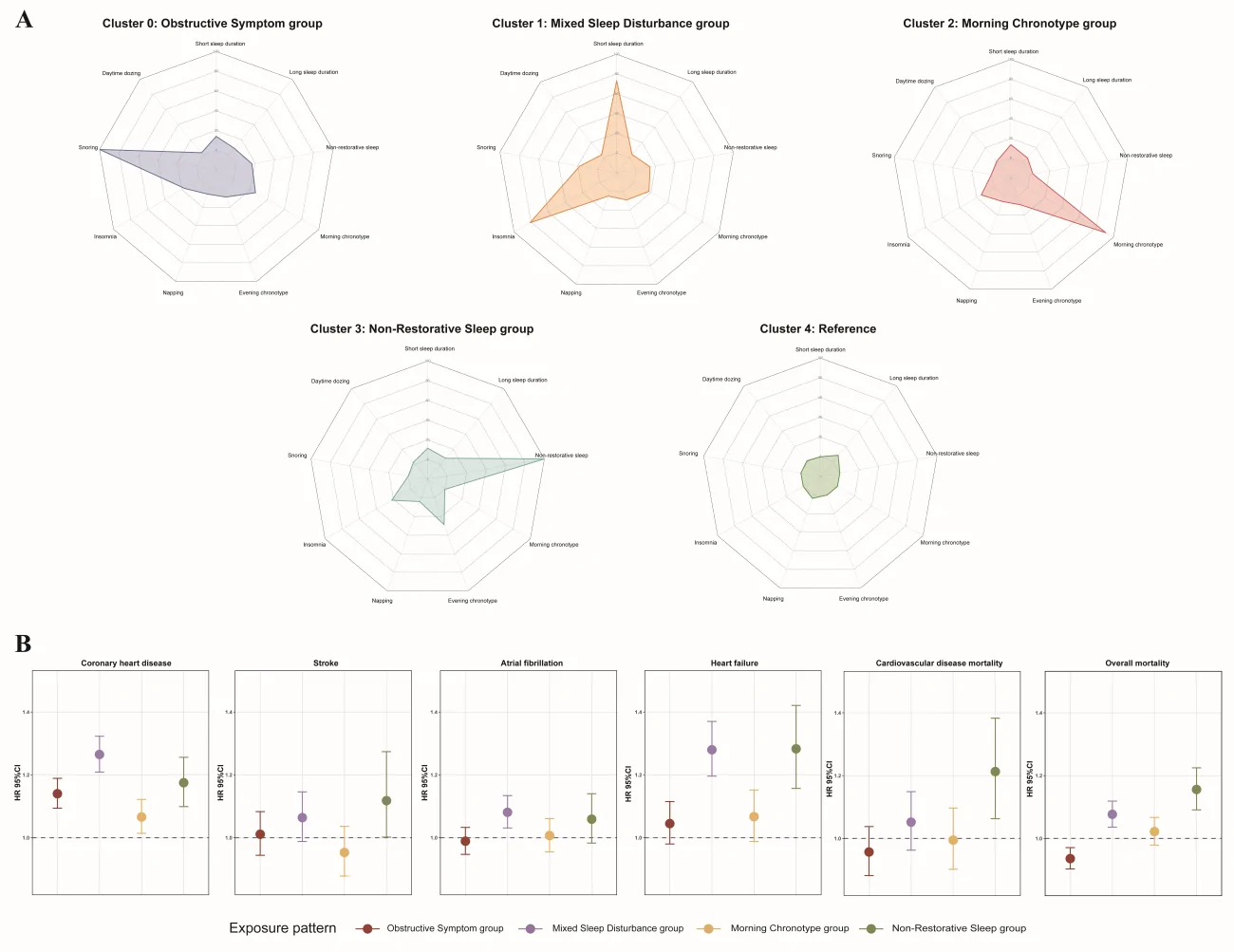
Sleep health patterns characteristics and effects on cardiovascular events and mortality. Points represent HR estimates, while vertical error bars denote 95% CIs. The horizontal dashed line indicates the null value (HR = 1.0), with the reference group serving as the comparator. Statistical significance was assessed using two-sided Wald tests. **Panel A.** Radar plots illustrate the characteristics of clusters identified through K-prototypes clustering. Each axis represents the relative value of a distinct sleep-related characteristic within each pattern. **Panel B.** Associations between co-exposure patterns and cardiovascular outcomes and mortality were estimated using multivariable Cox proportional hazards regression, adjusted for age, sex, ethnicity, TDI, BMI, employment status, educational level, smoking status, alcohol consumption, physical activity, and family history of heart disease and stroke. BMI – body mass index, CI – confidence interval, TDI – Townsend deprivation index, HR – hazard ratio.

The identified sleep patterns exhibited distinct associations with cardiovascular events and mortality (**Figure 2**, Panel B). Compared with the reference group, the mixed sleep disturbance pattern was associated with higher risks of CHD (HR = 1.27; 95% CI = 1.21, 1.32), AF (HR = 1.08; 95% CI = 1.03, 1.13), HF (HR = 1.28; 95% CI = 1.22, 1.36), and overall mortality (HR = 1.08; 95% CI = 1.04, 1.12), while its associations with stroke and CVD mortality were not statistically significant. The non-restorative sleep pattern was also associated with elevated risks across multiple outcomes, including CHD (HR = 1.18; 95% CI = 1.10, 1.26), stroke (HR = 1.12; 95% CI = 1.00, 1.27), HF (HR = 1.28; 95% CI = 1.21, 1.36), CVD mortality (HR = 1.21; 95% CI = 1.06, 1.38), and overall mortality (HR = 1.16; 95% CI = 1.09, 1.23). In contrast, the obstructive symptom pattern was associated with a higher risk of CHD (HR = 1.14; 95% CI = 1.09, 1.19), but showed little evidence of association with most other endpoints and was inversely associated with overall mortality (HR = 0.94; 95% CI = 0.90, 0.97). The morning chronotype pattern was largely not associated with adverse outcomes, although we observed a modest increase in CHD risk (HR = 1.07; 95% CI = 1.01, 1.12). The sleep pattern associations were also generally consistent in competing-risk analyses and analyses excluding events within the first year of follow-up (Tables S8 and S9 in the **Online Supplementary Document**).

### Identifying proteins associated with multi-dimensional sleep health score

To explore potential biological pathways underlying the association between sleep health and adverse outcomes, we next examined plasma proteins associated with SHS in participants with available proteomic data from the UK Biobank Pharma Proteomics Project (Figures S5–8 and Tables S15–17 in the **Online Supplementary Document**. We observed no substantial differences compared with the main cohort, with all standardised mean differences <0.1. To further evaluate comparability beyond individual variable standardised mean differences, we fitted a multivariable logistic regression model to predict proteomic data availability using demographic, socioeconomic, lifestyle, family-history, and sleep-related variables. The model showed low discrimination, with a 10-fold cross-validated area under the curve of 0.518 (95% CI = 0.514, 0.521), indicating that the joint baseline profile had little ability to distinguish participants with proteomic data from those without proteomic data. After multivariable linear regression, 188 of 2911 proteins were significantly associated with SHS after Bonferroni correction. Among these, only 5 proteins were positively associated with SHS, including CA14, PAEP, APOF, CRISP3, and CCN5, while the remaining 183 proteins were inversely associated with SHS, with CPM, GUSB, IGSF9, GAST, and ERBB2 among the most strongly associated signals. The Gene Ontology and Kyoto Encyclopedia of Genes and Genomes enrichment analyses indicated that these 188 proteins were mainly involved in leukocyte migration, chemotaxis, immune and inflammatory responses, cytokine–cytokine receptor interaction, and cellular components related to the vesicle lumen, lysosome, and membrane microdomains. In sensitivity analyses using elastic net regression, 73 representative proteins were identified, among which 61 overlapped with those selected by LASSO.

### Mediation analysis

To address the limited size of the proteomic subset and to ensure that the exposure–outcome associations were present within the mediation analytic sample, we first re-evaluated the associations between SHS and all six outcomes among participants with available proteomic data (Figures S9 and 10, and Table S18 in the **Online Supplementary Document**). When SHS was modelled as a continuous variable, higher SHS was directionally associated with lower risks of all six outcomes, with significant associations after FDR correction for CHD, stroke, HF, and overall mortality. The associations with AF and CVD mortality were directionally consistent but did not reach FDR-adjusted significance, likely reflecting the smaller sample size and fewer events in the proteomic subset. This suggests that the core SHS–outcome associations were broadly reproduced in the proteomic subset, albeit with reduced precision for some outcomes. We then further evaluated whether the 61 representative proteins showed statistical mediation signals in the associations of SHS with these four outcomes, where 34 showed significant mediation signals. Tests of SHS × protein interaction terms showed that none of the 244 interaction terms remained significant after FDR correction, either within each outcome or across all interaction tests, suggesting no strong evidence of exposure-mediator interaction in the mediation-related Cox models. Functional enrichment analyses indicated that proteins with mediation signals were mainly involved in inflammation- and immune-related processes, including chemotaxis, cytokine signalling, receptor-ligand activity, and cell surface- and vesicle-related cellular components.

Among the proteins showing significant mediation signals, HGF, IGF2R, and FABP4 showed relatively recurrent mediation signals across multiple outcomes (**Figure 3**). HGF showed the largest point estimate for overall mortality, accounting for 49.03% (95% CI = 15.54, 72.97) of the association, and also showed a mediation signal for HF, with a mediation proportion of 26.80% (95% CI = 5.03, 49.45). IGF2R showed mediation signals for overall mortality, stroke, and CHD, with mediation proportions of 30.35% (95% CI = 5.94, 53.01), 25.66% (95% CI = 4.76, 48.31), and 23.85% (95% CI = 4.34, 46.50), respectively. FABP4 also showed mediation signals for overall mortality and CHD, with mediation proportions of 31.26% (95% CI = 6.18, 53.92) and 15.19% (95% CI = 2.53, 37.85), respectively. Overall mortality involved a larger number of mediating proteins, suggesting that the association between sleep health and mortality may involve a broader range of proteomic pathways. CHD also showed mediation signals through multiple proteins, including IGF2R, CPM, HGF, CCL15, and AMBP, many of which are involved in inflammatory responses, metabolic regulation, and tissue injury-related processes. By contrast, fewer significant mediating proteins were identified for stroke and HF, and the corresponding mediation proportions were generally smaller. Due to several mediation proportions having wide confidence intervals, these results should be interpreted as exploratory mediation signals rather than definitive evidence of causal mediation.

**Figure 3:**
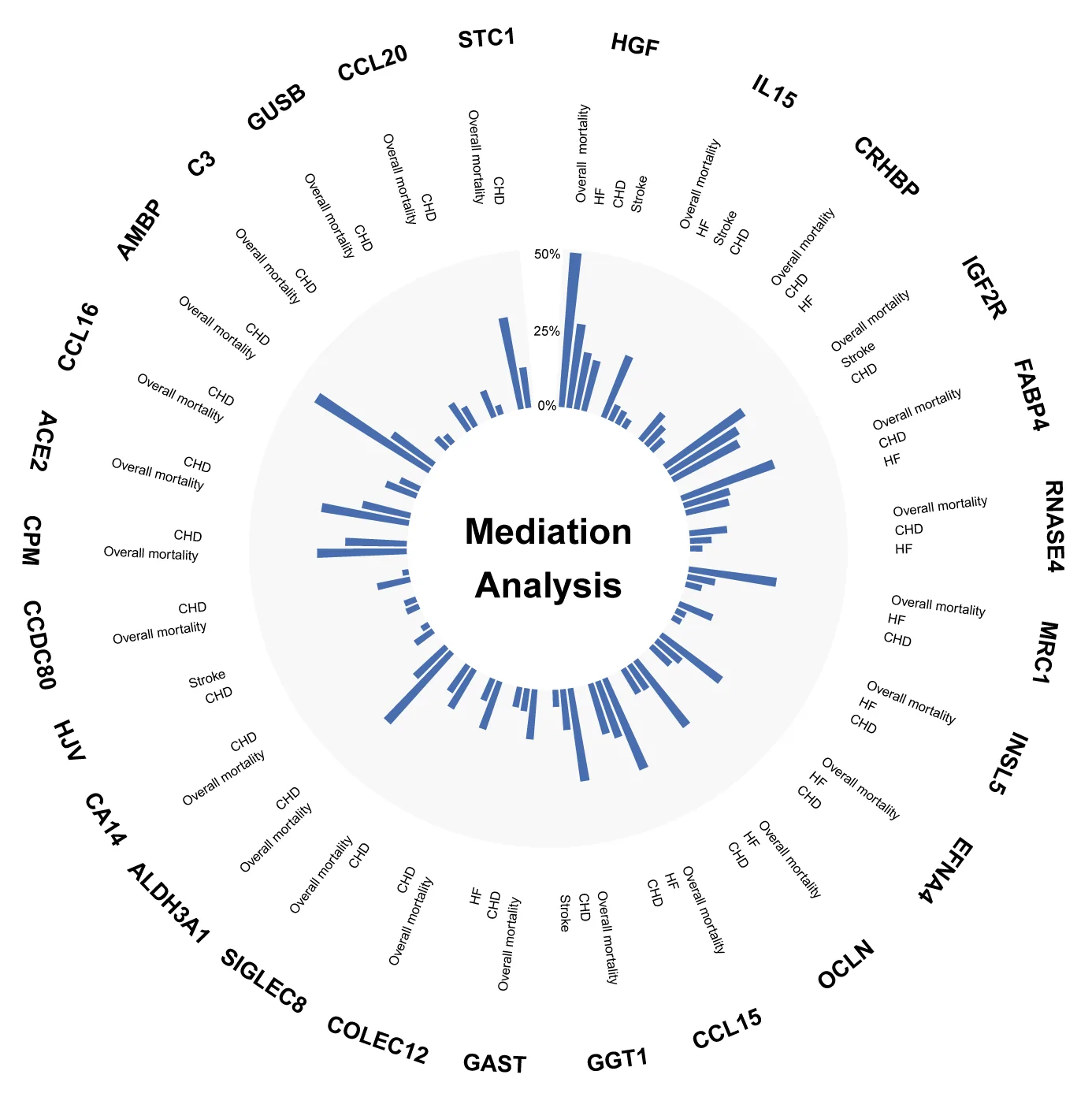
Exploratory mediation signals of proteins in the associations between the SHS and adverse health outcomes. This figure shows the estimated mediation proportions for 27 proteins showing exploratory mediation signals in at least two associations between the SHS and adverse health outcomes. The circular bar plot displays estimated mediation proportions derived from exploratory difference-in-coefficients analyses. Mediation analyses were adjusted for age, sex, ethnicity, TDI, BMI, employment status, educational level, smoking status, alcohol consumption, physical activity, and family history of heart disease and stroke. Two-sided *P*-values were FDR-corrected. ACE2 – angiotensin-converting enzyme 2, ALDH3A1 – aldehyde dehydrogenase, dimeric NADP-preferring, AMBP – alpha-1-microglobulin/bikunin precursor, BMI – body mass index, C3 – complement C3, CA14 – carbonic anhydrase 14, CCDC80 – coiled-coil domain-containing protein 80, CCL15 – C-C motif chemokine 15, CCL16 – C-C motif chemokine 16, CCL20 – C-C motif chemokine 20, CHD – coronary heart disease, COLEC12 – collectin-12, CPM – carboxypeptidase M, CRHBP – corticotropin-releasing factor-binding protein, EFNA4 – ephrin-A4, FABP4 – fatty acid-binding protein 4, adipocyte, GAST – gastrin, GGT1 – glutathione hydrolase 1 proenzyme, GUSB – beta-glucuronidase, FDR – false discovery rate, HF – heart failure, HGF – hepatocyte growth factor, HJV – hemojuvelin, IGF2R – insulin-like growth factor 2 receptor, IL15 – interleukin-15, INSL5 – insulin-like peptide INSL5, MRC1 – macrophage mannose receptor 1, OCLN – occludin, RNASE4 – ribonuclease 4, SIGLEC8 – sialic acid-binding Ig-like lectin 8, STC1 – stanniocalcin-1, TDI – Townsend deprivation index.

## DISCUSSION

In this prospective study based on data from the UK Biobank, we found that poorer multidimensional sleep health, whether assessed using the *a priori* SHS or data-driven sleep clustering patterns, was associated with a higher risk of cardiovascular events and mortality. Proteomic profiling identified 188 SHS-related proteins among 2,911 measured proteins, with enrichment mainly in inflammatory, immune, and chemotactic pathways. Exploratory mediation analyses further identified 34 proteins with potential mediation signals between sleep health and adverse outcomes, among which HGF, IGF2R, and FABP4 showed recurrent cross-outcome signals. The predictive information carried by SHS-related proteins was concentrated in a small number of key markers, particularly TNFRSF10B, HAVCR1, CXCL17, and ERBB3. Protein models showed lower discrimination than demographic models, indicating limited independent predictive utility of the selected proteomics group. Compared with demographic models, the combination model only showed moderate improvement, indicating that plasma proteins may provide complementary rather than alternative predictive information, particularly for HF and overall mortality.

The SHS analyses showed that as sleep health worsened, the risks of cardiovascular events and mortality increased overall in a dose-response manner, with the strongest associations seen for HF, CHD, and overall mortality. Cluster analyses further revealed marked heterogeneity across sleep phenotypes. Mixed sleep disturbance and non-restorative sleep patterns showed the most consistent associations with elevated risk across most outcomes, while the obstructive symptom pattern showed weaker associations, and the morning chronotype pattern was not clearly associated with increased risk overall. Taken together, these findings suggest that what is most relevant to cardiovascular risk is often not a single isolated sleep characteristic, but rather the accumulation and combination of multiple adverse sleep dimensions that together reflect an overall state of sleep dysregulation. These findings may also help explain why prior studies have often reported inconsistent associations between sleep and cardiovascular risk. Previous research has largely focused on single sleep dimensions, such as sleep duration, insomnia symptoms, snoring, daytime sleepiness, or chronotype. Although these studies generally support an association between abnormal sleep and risk of cardiovascular events and mortality, the direction and magnitude of effect have not been entirely consistent, suggesting that single-dimension measures may not adequately capture the true burden of sleep-related risk [28,29]. Sleep duration provides a typical example: its association with cardiovascular outcomes is often J-shaped or U-shaped, and duration alone is unlikely to explain the substantial heterogeneity in risk across individuals [29]. By contrast, a multidimensional sleep framework simultaneously captures information on sleep duration, quality, restorative value, circadian preference, and related functional impairment, and therefore more closely reflects sleep as a systemic health phenotype [9,30]. Chronotype may be harder to capture in a simplified score than other sleep dimensions. In our SHS, intermediate chronotype was assigned one point, while morning and evening chronotypes were assigned zero points. This coding does not mean that morning chronotype was considered harmful, but rather that the intermediate chronotype was used as the most neutral reference category when constructing the composite score. Consistent with this interpretation, the cluster analyses did not show a broadly adverse risk profile for the morning chronotype pattern. This suggests that the health implications of chronotype may depend more on the overall combination of sleep characteristics than on chronotype alone. Together with our findings, this body of evidence supports the view that sleep is better understood as a systems-level health phenotype rather than as a set of isolated single exposures [10].

The associations we observed here are biologically plausible. Experimental studies have shown that prolonged sleep deprivation disrupts autonomic and hypothalamic-pituitary-adrenal axis homeostasis and induces systemic inflammatory responses [31]. Chronic sleep fragmentation and intermittent hypoxia may further promote hypertension, arterial stiffness, endothelial dysfunction, and vascular remodelling [32,33]. Furthermore, insufficient sleep, irregular sleep, insomnia, and sleep-disordered breathing may contribute to the development of CHD, HF, AF, and stroke through shared pathways involving sympathetic overactivation, metabolic dysregulation, inflammation, and endothelial injury [34]. Therefore, the coexistence of short sleep, insomnia, non-restorative sleep, and daytime functional impairment may indicate a more severe and sustained state of biological dysregulation rather than simply poor sleep quality. This may explain why the mixed sleep disturbance and non-restorative sleep patterns showed stronger associations with CHD and HF than the obstructive symptom pattern, which mainly reflected a single symptom dimension. Similarly, previous work suggests that insomnia accompanied by short sleep duration represents a more biologically severe phenotype linked to adverse cardiovascular outcomes [35].

Although sleep health is closely related to cardiovascular events and mortality risk, its molecular basis remains incompletely understood. In our study, SHS-related proteins were mainly enriched in leukocyte migration, chemotaxis, immune-inflammatory responses, and cytokine–cytokine receptor interaction pathways, suggesting that the effects of poor sleep on cardiovascular events and mortality are unlikely to be driven by a single molecule. This interpretation is consistent with recent proteomic evidence in atherosclerosis [36]. These associations may involve interconnected biological processes related to immune-inflammatory activation, metabolic stress, and tissue injury and repair. Exploratory mediation analyses further highlighted HGF, IGF2R, and FABP4 as candidate proteins with recurrent cross-outcome mediation signals. Their relatively consistent mediating effects across different endpoints further suggest that the link between poor sleep health and adverse cardiovascular outcomes may involve processes related to injury and repair, growth factor signalling, and metabolic inflammation.

In risk prediction analyses, we further found that the predictive gain from proteomics was concentrated in a relatively small number of high-information proteins, rather than in the cumulative contribution of large protein panels. This pattern is consistent with recent large-scale population proteomics studies showing that proteomic information can add meaningful value to risk assessment, but that its predictive performance is often driven by a limited number of key markers rather than fully replacing conventional clinical and demographic predictors [37]. Notably, TNFRSF10B, HAVCR1, and CXCL17 were repeatedly identified as important features in the ML models and also appeared among the significant mediators of the association between SHS and CHD. This overlap suggests that these proteins may represent biologically relevant molecular signals related to poor sleep-associated cardiovascular risk, rather than merely data-driven statistical features.

Nevertheless, the protein-only model did not outperform the demographic and lifestyle model. This finding, too, is biologically plausible. Age, BMI, smoking, and physical activity are upstream and cumulative risk indicators that reflect long-term biological ageing and behavioural burden [38]. In contrast, plasma proteins measured at a single baseline time point represent more proximal molecular phenotypes: their circulating levels are shaped by genetic, biological, environmental, and technical factors, including modifiable risk factors, inflammatory status, and organ-function-related processes [39]. Therefore, plasma proteins may capture part of the downstream molecular imprint of long-term exposures such as aging, adiposity, smoking, and physical inactivity, but may not fully replace the broad and stable prognostic information carried by these upstream risk indicators. Furthermore, because we only measured protein levels only once at baseline, they may also be influenced by transient biological states and disease non-specificity. This does not diminish the biological value of proteomic profiling. Rather than serving as a replacement for established predictors, plasma proteins may provide complementary information on downstream molecular pathways, disease activity, and potential mediating mechanisms linking multidimensional sleep health to mortality risk [40].

This study has several strengths. First, it was based on the large-scale prospective UK Biobank cohort, with a substantial sample size and long follow-up, providing strong statistical power and a clear temporal framework for evaluating the associations of multidimensional sleep health with cardiovascular events and mortality. Second, we characterised sleep exposure using both the predefined SHS and data-driven sleep clustering patterns, allowing a more comprehensive representation of the coexistence and heterogeneity of sleep problems in the general population. Third, we integrated proteomic association analyses, mediation analyses, and ML prediction, thereby providing a multi-layered framework spanning epidemiologic associations, potential molecular pathways, and risk prediction. Finally, we performed both subgroup analyses to examine consistency across different strata and multiple sensitivity analyses, which supported the robustness of the main findings.

Several limitations, however, should be acknowledged. First, despite the prospective design and extensive covariate adjustment, the observational nature of this study precludes definitive causal inference, and residual confounding, unmeasured confounding, and reverse causation cannot be fully excluded. We were also unable to include some potentially relevant factors were in the used models, which may have influenced the observed associations. Second, the primary analyses were based on a complete-case cohort. Although we compared included and excluded participants and performed multiple-imputation and other sensitivity analyses that supported the robustness of the main findings, residual selection bias cannot be completely excluded, particularly because the seven-component SHS could not be reliably reconstructed for participants with missing sleep exposure data. Third, sleep health was assessed using self-reported sleep characteristics at baseline, which may be subject to recall error, reporting bias, and misclassification. Moreover, a single baseline assessment cannot capture changes in sleep health over time. Fourth, the mediation analyses should be interpreted as exploratory and hypothesis-generating rather than as definitive evidence of causal mediation. In addition, plasma protein levels may be influenced by diet, medication use, subclinical disease, inflammatory status, and sample handling or storage conditions, some of which may also be correlated with sleep health. Fifth, proteomic analyses were restricted to participants with available Olink measurements. Although baseline comparisons and assessment of proteomic data availability suggested that the proteomic subset was broadly comparable with the main analytic cohort, proteomic data availability may not be completely random, and unmeasured differences remain possible. The Olink platform also does not cover the entire circulating proteome, and measured protein levels may be affected by assay characteristics and platform-specific factors. Sixth, although the ML analyses used a split-first pipeline to minimise data leakage, the models were still developed and evaluated within the UK Biobank proteomic subset. Furthermore, the protein model did not outperform the demographic model, and the combined model provided only modest incremental improvement, suggesting limited standalone clinical utility of the selected proteomic panel. Therefore, external validation in independent cohorts is required before these protein panels can be considered clinically useful. Finally, because UK Biobank participants are predominantly White and relatively healthier and more socioeconomically advantaged than the general population, the generalisability of these findings requires caution. The seven-component SHS used equal weighting of sleep dimensions, and the prevalence, relative importance, and biological correlates of individual sleep components may differ across diverse populations. Future studies are needed to validate and, if necessary, recalibrate the SHS framework, proteomic mediation signals, and prediction models in more diverse and independent cohorts.

## CONCLUSIONS

We found that lower SHS was associated with higher risks of cardiovascular events and mortality. Proteomic analyses further suggested that these associations may involve plasma protein pathways related to inflammation, immunity, and chemotaxis, while exploratory mediation analyses identified several proteins with potential mediation signals. Among them, HGF, IGF2R, and FABP4 showed recurrent mediation signals across multiple outcomes, indicating that they may represent candidate biological links between sleep health and adverse cardiovascular outcomes. Although the predictive gains from ML were largely driven by a limited set of key markers, these findings suggest a potential biological basis underlying the relationship between sleep and cardiovascular health. They also underscore the importance of assessing sleep health from a multidimensional perspective and provide a foundation for future biomarker discovery, mechanistic validation, and precision prevention strategies targeting sleep-related cardiovascular risk.

## Additional material


Online Supplementary Document

## Data Availability

**Data availability:** The data used for analysis are available *via* the UK Biobank (https://www.ukbiobank.ac.uk/) under application number 969786.
