# Supplementary material for: Multidimensional sleep health: risk of cardiovascular disease and mortality, and mediation by plasma proteins
Source: J Glob Health. 2026 Oct 23;16:04349. doi: 10.7189/jogh.16.04349 (PMC13693298; doi:10.7189/jogh.16.04349)
Supplement: Supplementary file 1 [file jogh-16-04349-s001.zip › jogh-16-04349-s001.pdf]

Supplement to: Wei Z, Xiao Q, Wang S, Liu L, Meng D, Mao Z, Lu R, Hou R, Zhu X. Multidimensional sleep health: risk of cardiovascular disease and mortality, and mediation by plasma proteins. J Glob Health. 2026;16:04349.

Supplementary methods

Statistical analysis

Baseline characteristics were summarized across the SHS categories as median (interquartile range) for continuous variables and percentages for categorical variables. Group differences were assessed using the Kruskal-Wallis test for continuous variables and the Pearson's chi-square test or Fisher's exact test for categorical variables, as appropriate. Associations between SHS and risks of CVD and mortality were evaluated using multivariable Cox proportional hazards regression models, with results presented as hazard ratios (HRs) and 95% confidence intervals (CIs). The SHS was modeled as a continuous variable (per 1-point increase) and as a categorical variable, with the best sleep group (6-7 score) as the reference. Models were adjusted for age, sex, ethnicity, TDI, BMI, employment status, educational level, smoking status, alcohol consumption, physical activity, and family history of heart disease and stroke. Single-exposure multivariate Cox regression models were used to evaluate the association between individual sleep health factors and cardiovascular events and mortality. P values were adjusted using the Bonferroni correction. To further compare the relative contributions of individual factors, we used the eXtreme Gradient Boosting (XGBoost) model to calculate the SHapley Additive exPlanations (SHAP) values for each exposure. Furthermore, restricted cubic spline (RCS) models with three knots were applied to examine potential dose-response relationships between SHS and outcomes. To evaluate potential selection bias related to complete-case inclusion, we compared baseline characteristics between included and excluded participants and reported missingness separately for each variable. Differences were assessed using standardized mean differences (SMDs), with SMD > 0.1 considered to indicate potentially meaningful imbalance.

Stratified analyses were conducted to examine the associations of SHS with cardiovascular events and mortality according to age (> 60 years, ≤ 60 years) and sex

(male, female). Potential effect modification was assessed by including multiplicative interaction terms between SHS and the corresponding stratification variables in the full-sample models. P values for subgroup-specific associations and interaction terms were adjusted using the false discovery rate (FDR) method. Several sensitivity analyses were performed to assess the robustness of the findings. First, competing risk models were applied to account for the competing risk of death. Second, to reduce potential reverse causation, analyses were repeated after excluding participants who developed outcomes within the first year of follow-up. Third, to assess the influence of missing covariate data, we performed an additional sensitivity analysis using multiple imputation by chained equations with the mice package in R. Participants with missing or invalid sleep exposure data were not imputed because the seven-component SHS could not be reliably reconstructed without the original sleep information. Moreover, to further evaluate the robustness of the SHS definition, we constructed three alternative SHS measures. First, “sometimes” responses for insomnia symptoms, napping, and daytime dozing were classified as unfavorable. Second, chronotype was alternatively coded by treating morning and intermediate chronotypes as favorable and evening chronotypes as unfavorable. Third, chronotype was removed from the SHS, resulting in a six-component score. For each alternative SHS, we repeated the multivariable Cox regression analyses for all six outcomes using the same covariate adjustment as in the primary analysis.

K-prototypes clustering was applied to identify distinct sleep health patterns based on the seven sleep-related indicators included in the SHS. The K-prototypes algorithm combines K-means and K-modes, making it suitable for analyzing mixed categorical and continuous sleep variables and capturing inherent heterogeneity in sleep profiles [1]. The optimal number of clusters was determined using the elbow method in combination with the Calinski-Harabasz (CH) index, which plateaued at five clusters, indicating a balance between within-cluster homogeneity and between-cluster separation **(Figure S2)**. Clustering analyses were restricted to participants with complete exposure data. Associations between the identified sleep

health patterns and risks of cardiovascular events and mortality were evaluated using multivariable Cox proportional hazards regression models.

Associations between the SHS and plasma protein levels were examined using multivariable linear regression models, with each protein analyzed separately as the dependent variable and SHS as the independent variable. Proteins were standardized prior to analysis, and statistical significance was determined using Bonferroni correction across the 2,911 tested proteins. Proteins significantly associated with SHS were subsequently subjected to functional enrichment analyses, including Gene Ontology (GO) terms for biological process (BP), cellular component (CC), and molecular function (MF), as well as Kyoto Encyclopedia of Genes and Genomes (KEGG) pathways, with P values adjusted using the FDR method. To identify a parsimonious set of representative proteins, least absolute shrinkage and selection operator (LASSO) regression was applied with 10-fold cross-validation, and the optimal regularization parameter was selected based on lambda.min. Proteins exhibiting 100% selection frequency (non-zero coefficients in all 1,000 iterations) were retained for downstream analyses. As a supplementary analysis, Elastic Net regression was additionally performed to evaluate the robustness of protein selection, and overlap between proteins identified by the two methods was compared.

Mediation analyses were restricted to participants with available proteomic data. The comparability of the proteomic subset with the main analytic cohort was assessed using SMDs and a multivariable logistic regression model predicting proteomic data availability from demographic, socioeconomic, lifestyle, family-history, and sleep-related variables. Within the proteomic subset, SHS-outcome associations were re-evaluated using multivariable Cox models, and outcomes that remained significant after FDR correction were carried forward for mediation analysis. Candidate proteins were evaluated individually using a difference-in-coefficients approach on the log-HR scale [2]. For each protein, the total effect was estimated from a Cox model including the SHS and covariates, and the direct effect from a corresponding model additionally including the protein. The

indirect effect was calculated as the difference between the total and direct effects, and the mediation proportion as the indirect effect divided by the total effect. Bootstrap resampling was used to estimate confidence intervals, and FDR correction was applied for multiple testing. SHS × protein interaction terms were tested as supplementary analyses. Because sleep health and plasma proteins were both measured at baseline, mediation estimates were interpreted as exploratory statistical decomposition rather than definitive causal mediation. Proteins identified as significant mediators were subsequently subjected to GO enrichment analyses to explore the biological processes related to the observed mediation signals.

Machine learning (ML) analyses were performed to evaluate the predictive value of protein biomarkers using a leakage-minimized split-first framework. For each prediction task, participants were first randomly divided into training and test sets in an 8:2 ratio using stratified sampling according to outcome status. All subsequent steps, including protein screening, LASSO-based feature selection, Light Gradient Boosting Machine (LightGBM) model training, hyperparameter tuning, protein importance ranking, and sequential forward feature selection, were performed exclusively within the training set. Specifically, within the training set, proteins associated with the SHS were identified and entered into LASSO regression to select representative candidate proteins. These selected proteins were then entered into a LightGBM model, and protein importance was ranked according to its contribution to model performance. Predictive performance during model development was assessed using 10-fold cross-validation within the training set. Sequential forward feature selection was subsequently applied by incorporating proteins into the model one at a time according to their importance ranking. Sequential forward selection was stopped when adding additional proteins failed to yield significant incremental improvement in AUC in two consecutive DeLong tests [3]. After feature selection and model development were completed, the final selected protein panel and locked model were evaluated in the independent test set, which was held out and not used in any stage of protein screening, feature selection, model training, or model tuning. The predictive

performance of the protein model was compared with that of a demographic model and a combined protein + demographic model using area under the curve (AUC). The contributions of selected proteins to model predictions were further interpreted using SHAP values. Additionally, model performance was assessed across different time windows (5 years, 5–10 years, and over 10 years) to evaluate temporal robustness.

Supplementary results

Machine learning identifies proteins with predictive value

ML analyses further demonstrated that a limited set of SHS-related proteins captured substantial predictive information for subsequent cardiovascular events and mortality. As shown in **Figure S11**, the cumulative AUC for overall mortality increased steeply after the sequential inclusion of the top-ranked proteins and then approached a plateau, indicating that most of the discriminative gain was concentrated in a relatively small number of markers. Based on this procedure, TNFRSF10B, HAVCR1, CXCL17, ERBB3, CA14, and ADAMTS15 were retained in the final model. The SHAP plot showed that TNFRSF10B, HAVCR1, and CXCL17 contributed most prominently to model prediction, with higher levels of these proteins generally corresponding to a greater predicted risk of death. In the independent test set, the protein-based model achieved an AUC of 0.738 for overall mortality, which was lower than that of the demographic model (AUC = 0.768). The combined protein + demographic model yielded the highest discrimination, with an AUC of 0.784, indicating a modest incremental improvement over the demographic model alone. When prediction was stratified by follow-up duration, the protein model maintained broadly comparable performance across different time windows, with AUC of 0.723

for events occurring within 5 years, 0.712 for those occurring between 5 and 10 years, and 0.728 for those occurring after 10 years, supporting the temporal stability of the selected protein panel.

Similar patterns were observed for cardiovascular outcomes (**Figure S11**). A small subset of proteins accounted for most of the predictive signal, particularly TNFRSF10B, HAVCR1, CXCL17, and ERBB3, repeatedly ranked among the leading contributors across different endpoints. The protein model of HF showed relatively strong discriminative ability (AUC = 0.764), which approached that of the demographic model (AUC = 0.786) and improved further after integration with demographic variables (AUC = 0.806). For CHD and stroke, the addition of protein markers to demographic variables also led to modest improvements in prediction performance, with the combined models outperforming either component model alone. By contrast, the incremental gain for AF was limited, although the same core proteins remained prominent contributors. Time-window analyses for cardiovascular endpoints yielded generally consistent results, indicating that the predictive value of these proteins was not restricted to short-term events. Although protein-only models were generally not superior to demographic models, the incorporation of proteomic markers modestly but consistently improved discrimination for major outcomes, with the most notable improvement observed for HF. The recurrent selection of TNFRSF10B, HAVCR1, and CXCL17 across multiple endpoints further supports their potential importance as key proteomic correlates of adverse prognosis.

1. Huang Z. Extensions to the k-means algorithm for clustering large data sets with categorical values. 1998, 2:283-304.

2. Li R, Tian S, Liu J, Li R, Zhu K, Lu Q, Qiu Z, Yu H, Li L, O HF, et al: Modifiable risk factors and plasma proteomics in relation to complications of type 2 diabetes. Nat Commun 2025, 16:2896.

3. Guo Y, You J, Zhang Y, Liu WS, Huang YY, Zhang YR, Zhang W, Dong Q, Feng JF, Cheng W, Yu JT: Plasma proteomic profiles predict future dementia in healthy adults. Nat Aging 2024, 4:247-260.

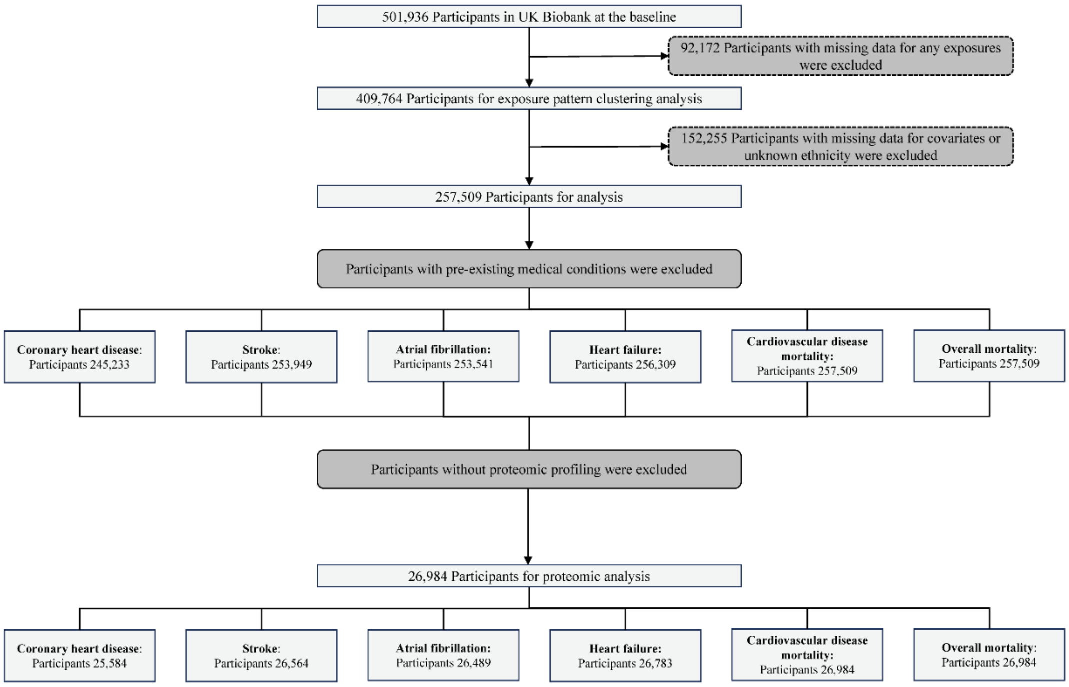

Figure S1. Participant inclusion and exclusion flowchart in the UK Biobank.

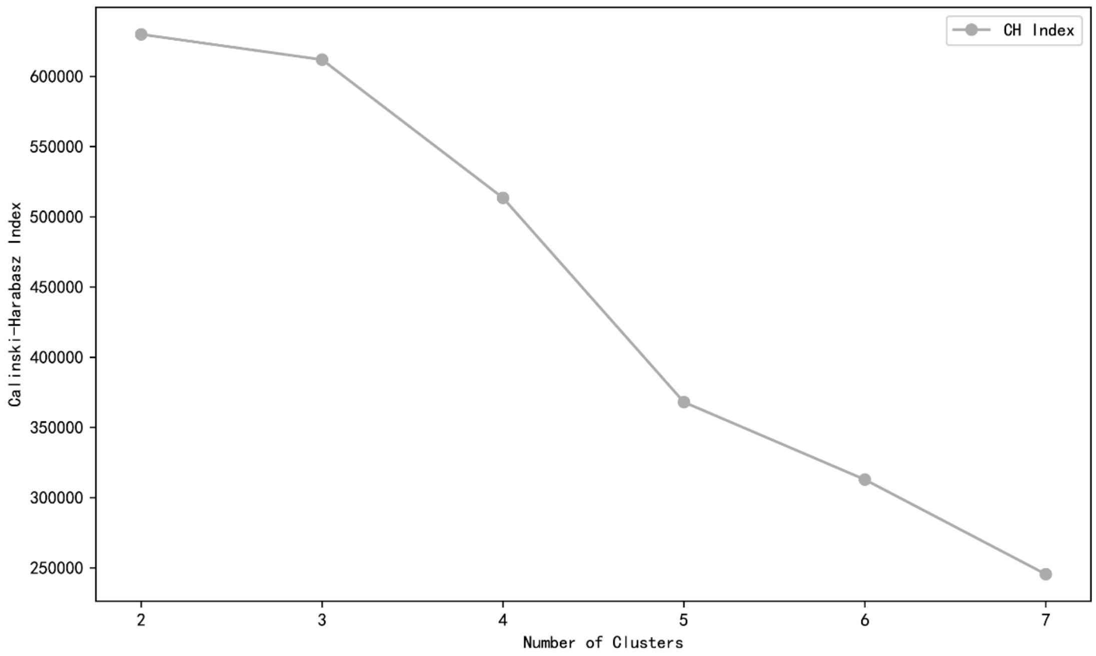

Figure S2. CH-index across different cluster numbers in K-prototypes analysis.

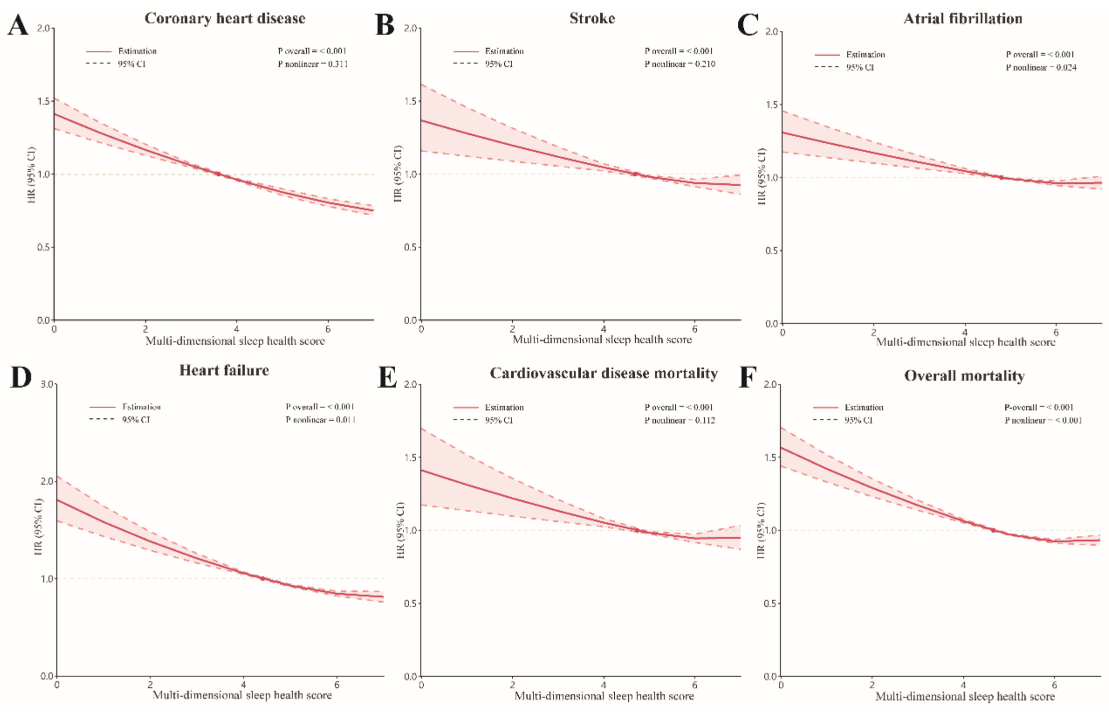

Figure S3. Associations of the SHS With Risks of Cardiovascular Events and Mortality.

The x-axis shows the SHS, and the y-axis represents hazard ratios (HRs). The solid red lines indicate the estimated HRs, and the light red shaded areas represent 95% confidence intervals derived from restricted cubic spline models with three knots. The models were adjusted for age, sex, ethnicity, Townsend deprivation index, body mass index, employment status, educational level, smoking status, alcohol consumption, physical activity, and family history of heart disease and stroke. All P values were based on two-sided tests.

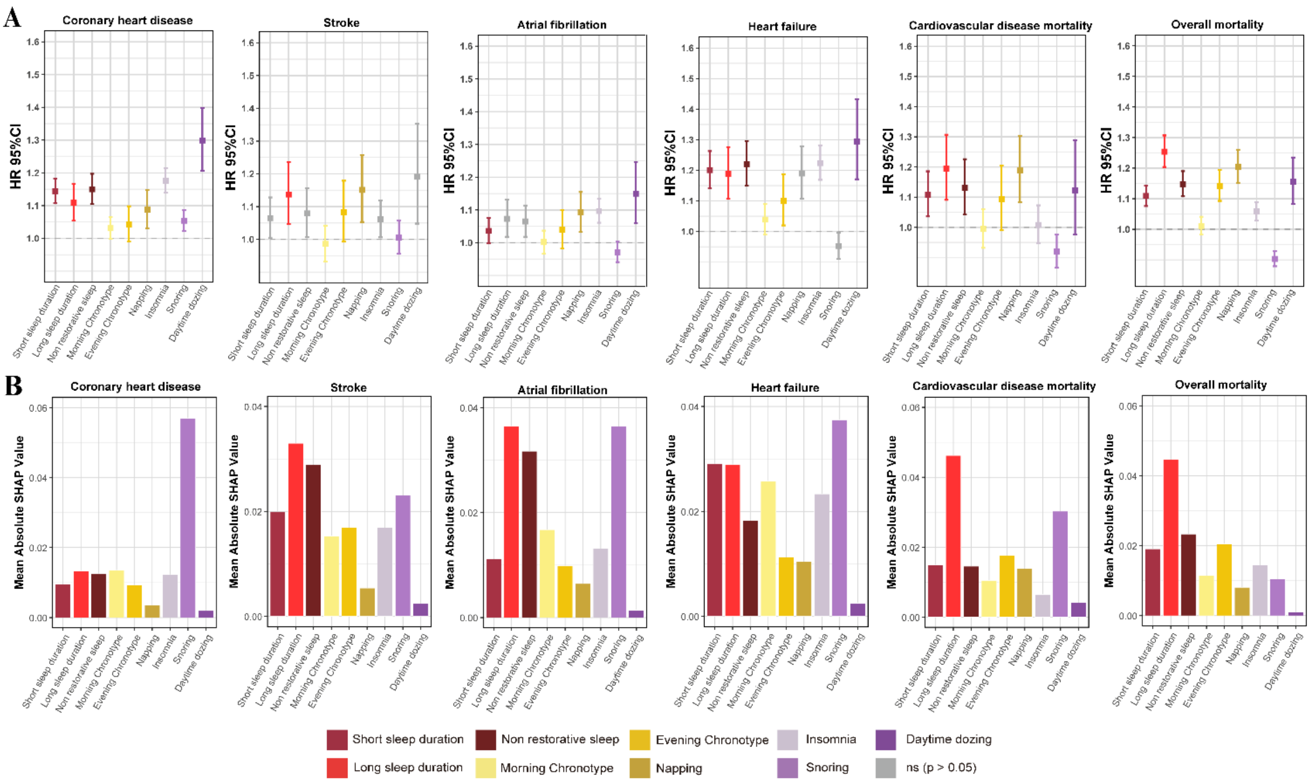

Figure S4. Individual sleep health factors and risks of cardiovascular outcomes and mortality.

A Hazard ratios (HRs) were estimated using single-exposure multivariable Cox proportional hazards models, adjusted for age, sex, ethnicity, Townsend deprivation index, body mass index, employment status, educational level, smoking status, alcohol consumption, physical activity, and family history of heart disease and stroke. All exposures were standardized before modeling. Circular markers represent HR point estimates for the association between each exposure and outcome, and vertical error bars indicate 95% confidence intervals (CIs). The horizontal dashed line denotes the null value (HR = 1). Colors represent different exposure types, with gray indicating exposures with adjusted P > 0.05. Statistical significance was assessed using two-sided Wald tests with Bonferroni correction for multiple comparisons. **B** Relative importance of each standardized sleep health factor for cardiovascular outcomes and mortality, based on mean absolute Shapley Additive exPlanations (SHAP) values derived from XGBoost models.

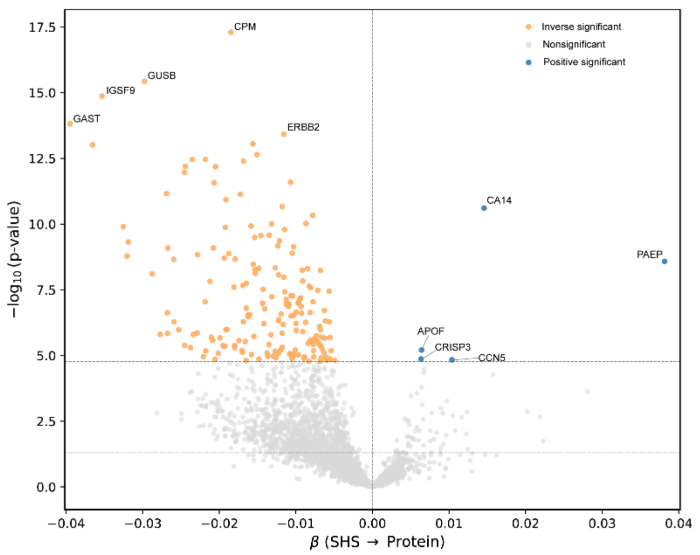

Figure S5. Volcano plot of associations between the SHS and 2,911 proteins. The x-axis shows the regression coefficient (β) for the association between the SHS and each protein, and the y-axis shows statistical significance (-log10 p-values). Blue and orange dots represent Bonferroni-significant positive and inverse associations, respectively, whereas gray dots represent non-significant associations. Statistical significance was defined as a Bonferroni-corrected P < 0.05. Multiple linear regression model was adjusted for age, sex, ethnicity, Townsend deprivation index, body mass index, employment status, educational level, smoking status, alcohol consumption, physical activity, and family history of heart disease and stroke. SHS, sleep health score; CA14, carbonic anhydrase 14; PAEP, glycodelin; APOF, apolipoprotein F; CRISP3, cysteine-rich secretory protein 3; CCN5, CCN family member 5; CPM, carboxypeptidase M; GUSB, beta-glucuronidase; IGSF9, immunoglobulin superfamily member 9; GAST, gastrin; ERBB2, receptor tyrosine-protein kinase erbB-2.

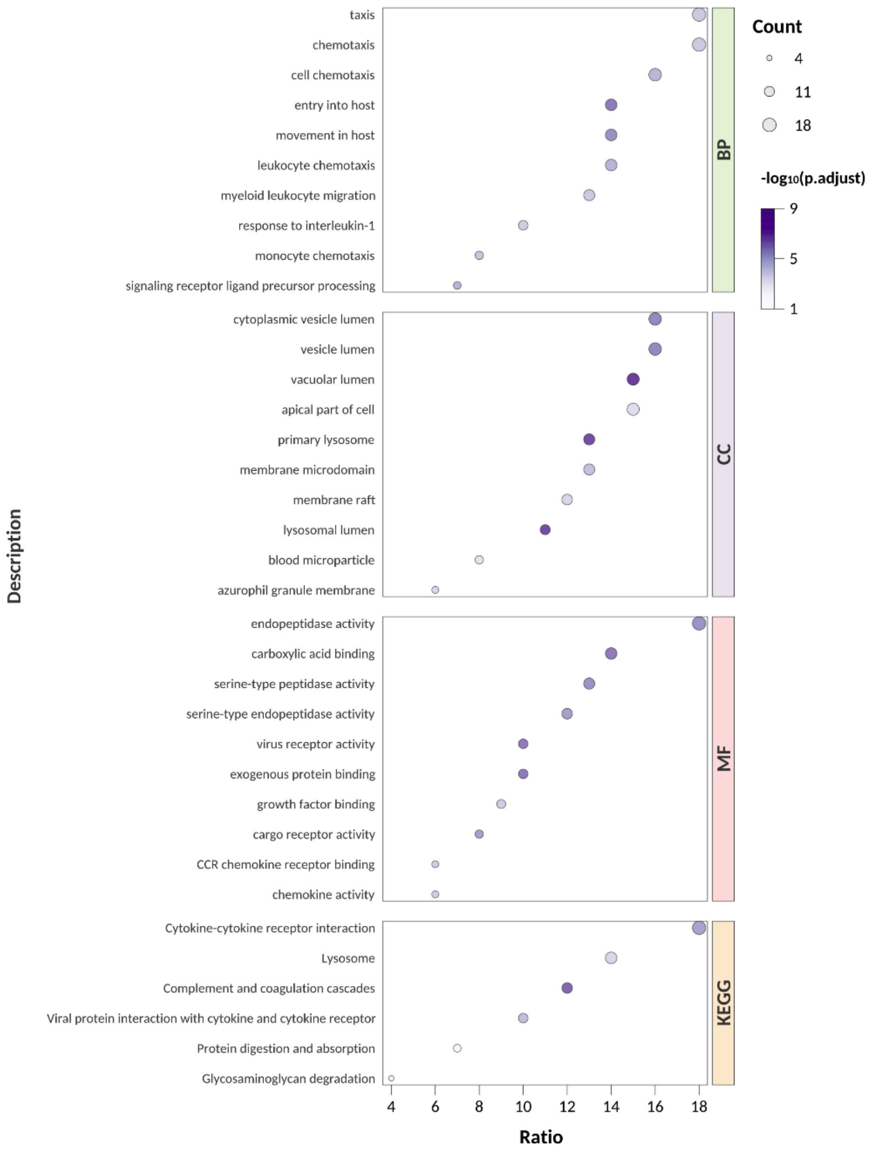

Figure S6. Enriched GO terms and KEGG pathways for 188 proteins significantly associated with the SHS.

Dot plots show the top 10 significantly enriched Gene Ontology (GO) terms for biological process (BP), cellular component (CC), and molecular function (MF), as well as the top 5 significantly enriched Kyoto Encyclopedia of Genes and Genomes (KEGG) pathways. The x-axis shows the enrichment ratio, and the y-axis lists the enriched pathways. Dot size represents the number of proteins, and dot color indicates statistical significance. P values were based on two-sided tests, and an FDR-corrected p value < 0.05 was considered statistically significant.

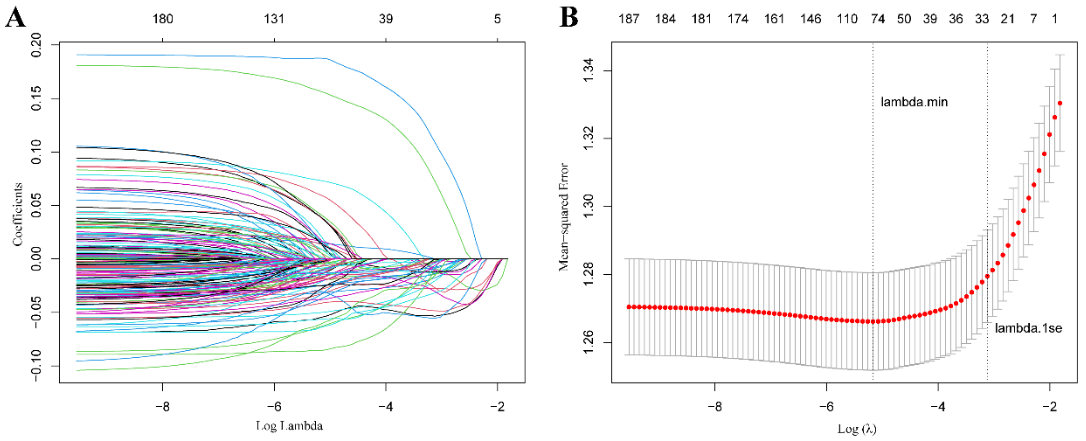

Figure S7. Candidate protein selection by LASSO regression.

A The coefficients of the independent variables in LASSO regression, based on the value of lambda. **B** Use lambda.min to select the optimal model. The model corresponding to lambda.min typically provides the best fit. The model corresponding to lambda.1se minimizes the number of variables while maintaining a relatively high level of fit. 10-fold cross-validation helps determine the optimal LASSO regularization parameter lambda.min and select proteins from the best-performing model. To prevent overfitting and improve stability, the LASSO regression was repeated 1,000 times.

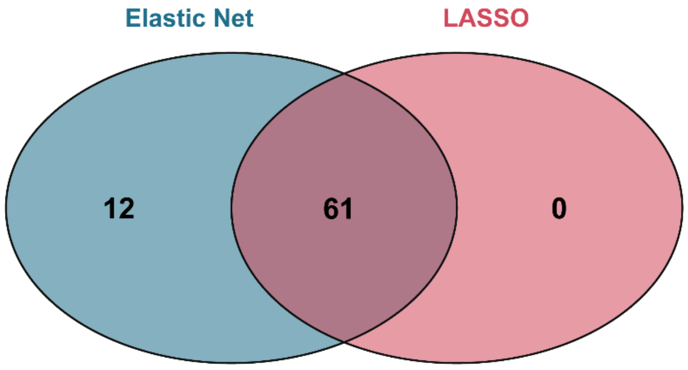

Figure S8. Venn diagram of proteins selected by Elastic Net and LASSO regression.

The blue circle indicates proteins selected by Elastic Net, and the red circle indicates proteins selected by LASSO. The overlap represents proteins identified by both methods, and the numbers indicate protein counts. Twelve proteins were additionally identified by Elastic Net only: ADAMTSL2, BST2, DDAH1, FGF21, FUOM, MEGF9, TOMM20, VNN1, COL18A1, LGALS4, PTS, and UPB1. ADAMTSL2, ADAMTS-like protein 2; BST2, bone marrow stromal antigen 2; DDAH1, dimethylarginine dimethylaminohydrolase 1; FGF21, fibroblast growth factor 21; FUOM, fucose mutarotase; MEGF9, multiple epidermal growth factor-like domains protein 9; TOMM20, mitochondrial import receptor subunit TOM20 homolog; VNN1, pantetheinase; COL18A1, collagen alpha-1(XVIII) chain; LGALS4, galectin-4; PTS, 6-pyruvoyl tetrahydrobiopterin synthase; UPB1, beta-ureidopropionase.

Outcome	Events/person-years	HR (95% CI)	P_{FDR}*
Coronary heart disease	2,102/394,304	0.92 (0.89, 0.96)	<0.001
Stroke	770/406,296	0.92 (0.87, 0.99)	0.027
Atrial fibrillation	1,812/398,711	0.96 (0.92, 1.00)	0.075
Heart failure	1,069/405,892	0.90 (0.85, 0.95)	<0.001
CVD mortality	628/409,095	0.93 (0.86, 1.01)	0.082
Overall mortality	2,987/395,439	0.95 (0.92, 0.99)	0.010

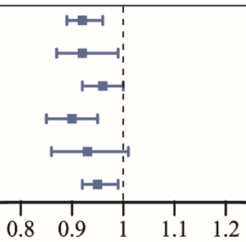

Figure S9. Associations of the SHS with risks of cardiovascular events and mortality among participants with available proteomic data.

The blue squares represent estimated hazard ratios (HRs), and horizontal error bars indicate 95% confidence intervals (CIs). The vertical dashed line denotes the null value (HR = 1.0). HRs were estimated using multivariable Cox proportional hazards regression models adjusted for age, sex, ethnicity, Townsend deprivation index, body mass index, employment status, educational level, smoking status, alcohol consumption, physical activity, and family history of heart disease and stroke. *P values were based on two-sided tests and were adjusted using the false discovery rate (FDR) method.

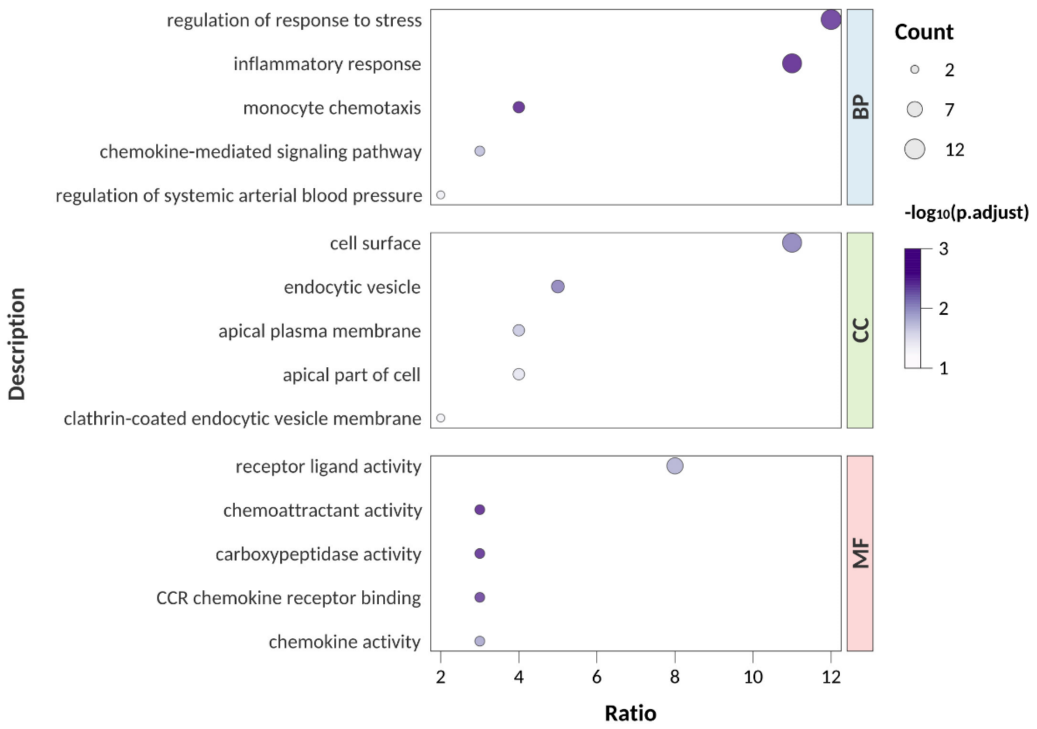

Figure S10. Enriched GO terms for 34 proteins showing exploratory mediation signals in the associations between the SHS and at least one outcome.

Dot plots show the top 5 significantly enriched Gene Ontology (GO) terms for biological process (BP), cellular component (CC), and molecular function (MF) among the 34 proteins. P values were based on two-sided tests, and an FDR-corrected p value < 0.05 was considered statistically significant.

Coronary heart disease:

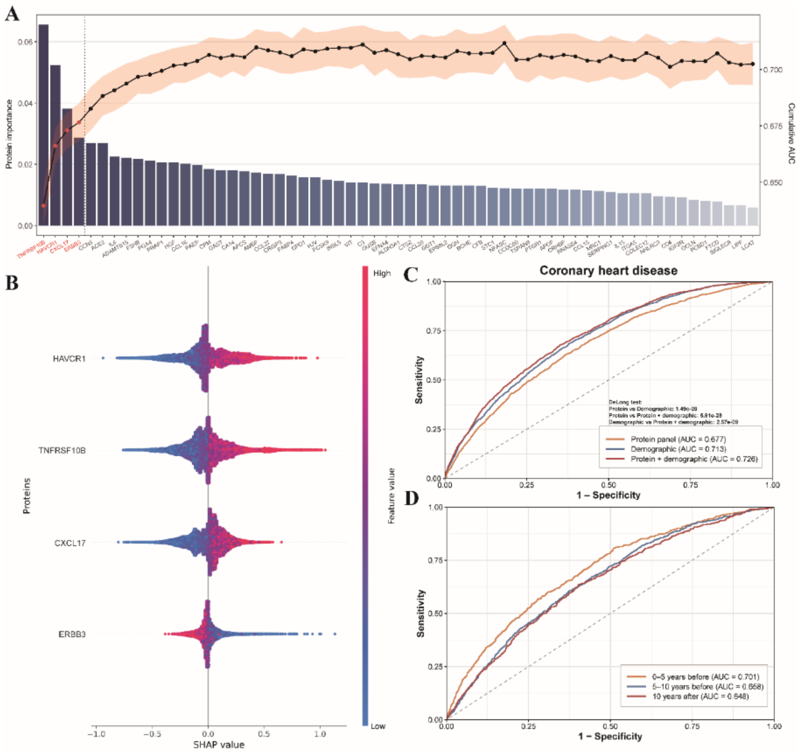

Stroke:

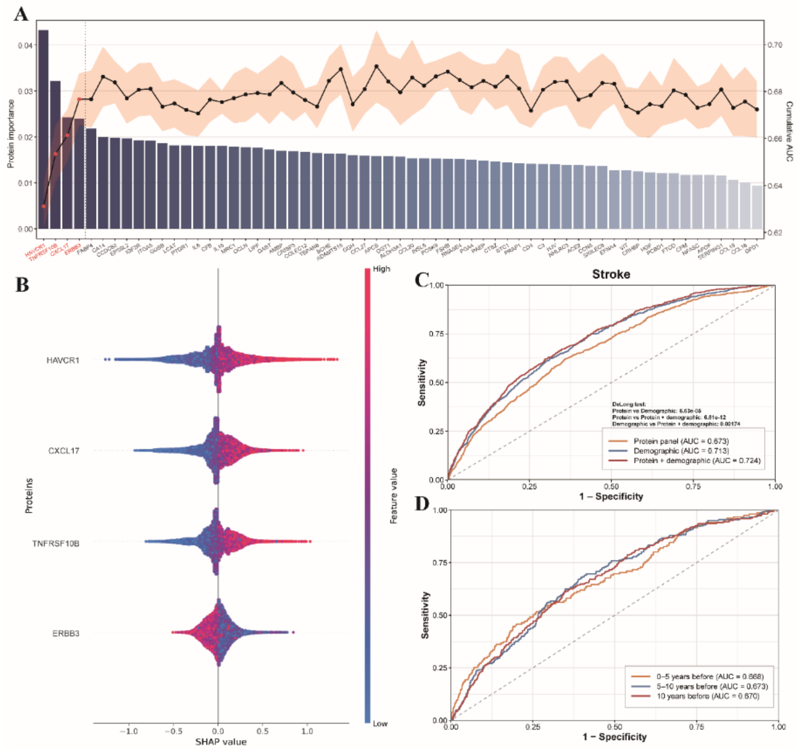

Atrial fibrillation:

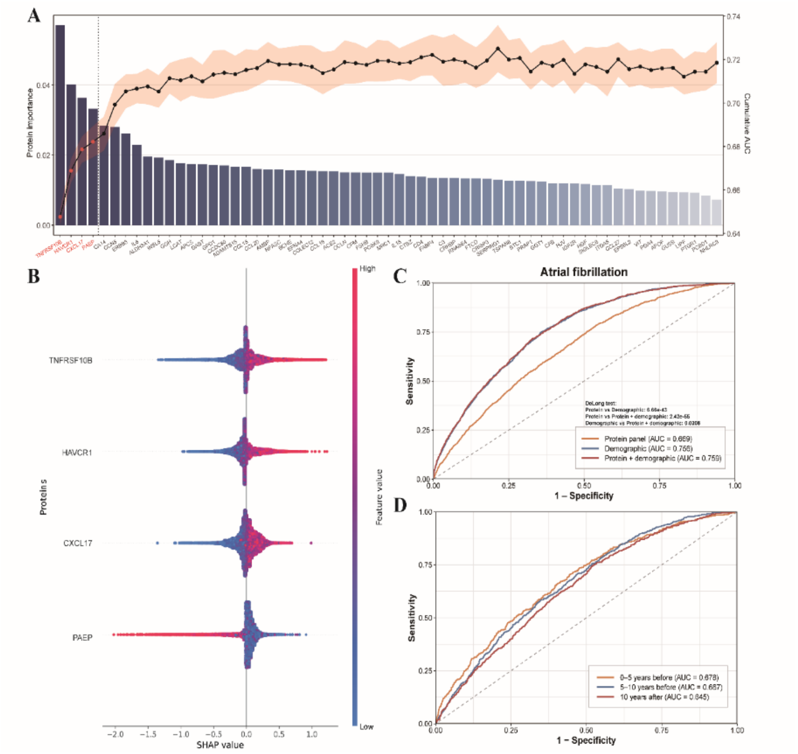

Heart failure:

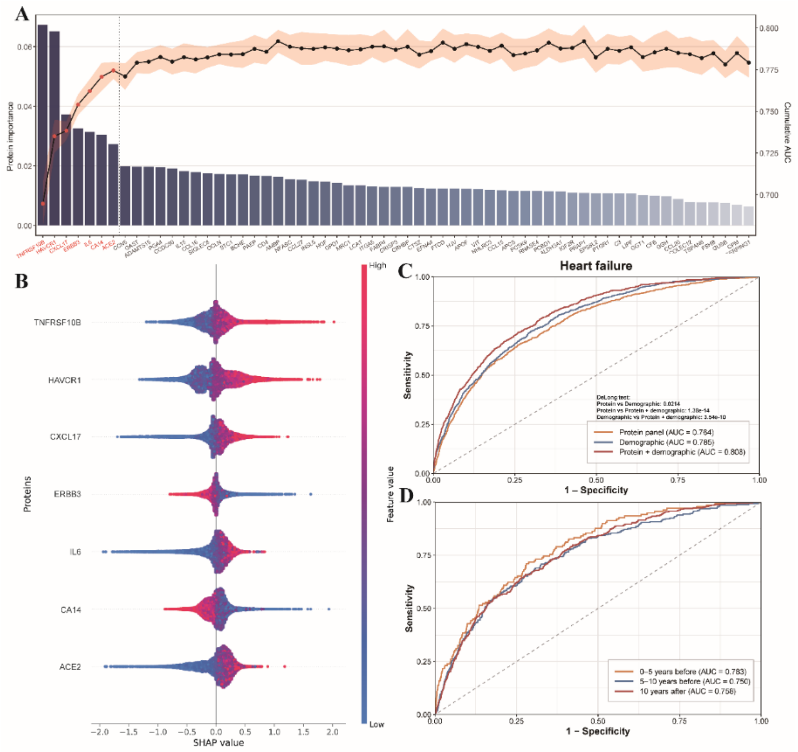

Cardiovascular disease mortality:

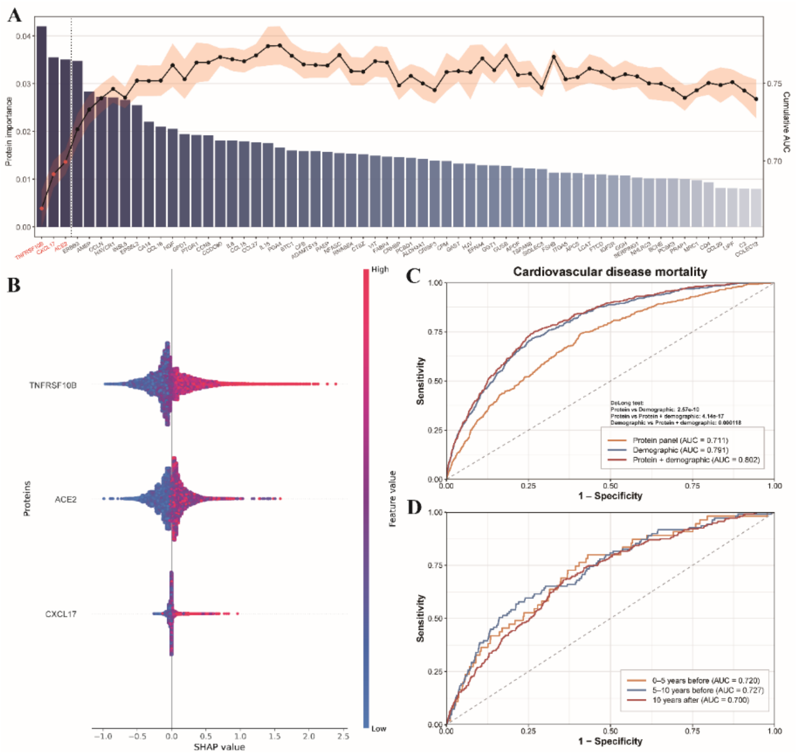

Overall mortality:

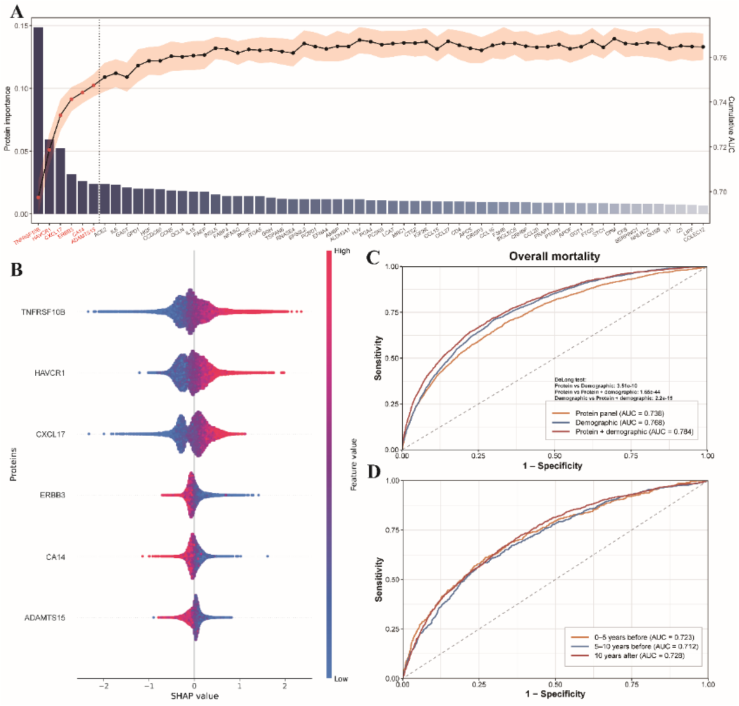

Figure S11. Prediction of cardiovascular disease and mortality using SHS-related proteins.

A Ranking of plasma protein feature importance for predicting future cardiovascular disease and mortality. The bar chart shows the importance of the training-set-derived LASSO-selected candidate proteins according to their contributions to prediction, as measured by information gain. The line plot indicates the cumulative area under the receiver operating characteristic curve (right

axis) as proteins were added sequentially during forward feature selection. Proteins ultimately retained in the final model are highlighted in red. Shaded areas represent standard errors derived from cross-validation. **B** SHAP summary plot of selected proteins. Color indicates protein abundance from low (blue) to high (red), and SHAP values indicate the direction and magnitude of contribution to model prediction. **C** Performance of the protein-based model, compared with the demographic model and the combined protein + demographic model for predicting cardiovascular events. **D** Performance of the protein-based model for predicting cardiovascular events across different time windows in the test dataset, including 0 – 5 years, 5 – 10 years, and > 10 years.

Supplementary Table 1. Adherence to JoGH's GRABDROP guidelines items.

JoGH guideline item	Author's Response
1. Please list all papers published by each co-author in previous 3 years that were based on secondary analysis of a big data repository	1. Wang F, Guo Y, Tang Y, Zhao S, Xuan K, Mao Z, Lu R, Hou R, Zhu X. Combined assessment of stress hyperglycemia ratio and glycemic variability to predict all-cause mortality in critically ill patients with atherosclerotic cardiovascular diseases across different glucose metabolic states: an observational cohort study with machine learning. Cardiovasc Diabetol. 2025 May 9;24(1):199. doi: 10.1186/s12933-025-02762-0<br>2. Sun Y, Guo Y, Ma S, Mao Z, Meng D, Xuan K, Lu R, Pan X, Zhu X. Association of C-reactive protein-triglyceride glucose index with the incidence and mortality of cardiovascular disease: a retrospective cohort study. Cardiovasc Diabetol. 2025 Aug 1;24(1):313. doi: 10.1186/s12933-025-02835-0<br>3. Tang Y, Gao X, Wang B, Kong L, Fan Y, Zhu X. Depressive disorder in middle-aged and elderly adults: Evidence from global burden of disease and genetic analysis. J Affect Disord. 2026 Mar 1;396:120766. doi: 10.1016/j.jad.2025.120766
2. Please explain the key elements of your study design and the use of the available datasets that make your study an original scientific contribution	Based on a large-scale prospective cohort from the UK Biobank, including 257,509 participants in the primary analysis and 26,984 participants with available plasma proteomic data, we systematically examined the association between multidimensional sleep health and incident cardiovascular events and mortality. The originality of this study is reflected in several aspects. First, instead of focusing on a single sleep trait, we assessed sleep health using a seven-component sleep health score and data-driven K-prototypes clustering, which allowed us to capture both the cumulative burden and the real-world co-occurrence patterns of multiple sleep characteristics. Second, we evaluated several clinically important outcomes, including coronary heart disease, stroke, atrial fibrillation, heart failure, cardiovascular mortality, and overall mortality. Third, we integrated plasma

proteomic profiling to identify sleep-health-related proteins and biological pathways. Finally, mediation analysis, and machine-learning prediction were combined to explore potential biological links, and predictive value. To our knowledge, no previous UK Biobank study has jointly integrated multidimensional sleep phenotyping, cardiovascular and mortality outcomes, plasma proteomics, mediation analysis, and machine-learning prediction in this context.

3. Please list all publications that addressed similar research questions in the same dataset and indicate where you cited them in your paper

We conducted a literature review and identified several UK Biobank studies that addressed related, but not identical, research questions. These studies were cited in the revised manuscript to contextualize our findings.

1.Tao F, Cao Z, Jiang Y, et al. Associations of sleep duration and quality with incident cardiovascular disease, cancer, and mortality: a prospective cohort study of 407,500 UK Biobank participants. Sleep Med. 2021;81:401–409.

2.Kianersi S, Potts KS, Wang H, et al. Chronotype, Life's Essential 8, and risk of cardiovascular disease: a prospective cohort study in UK Biobank. J Am Heart Assoc. 2026;15:e044189.

3.Huang T, Beydoun MA, Kianersi S, Redline S, Launer LJ. Multidimensional sleep health and dementia risk: a prospective study in the UK Biobank. BMC Med. 2025;23:410.

4.Zhang S, Zhang M, Yuan Y, et al. Health risks and genetic architecture of objectively measured multidimensional sleep health. Nat Commun. 2025;16:7026.

5.Sun BB, Chiou J, Traylor M, et al. Plasma proteomic associations with genetics and health in the UK Biobank. Nature. 2023;622:329–338.

6.You J, Guo Y, Zhang Y, et al. Plasma proteomic profiles predict individual future health risk. Nat Commun. 2023;14:7817.

4. Please explain how you addressed multiple testing through an appropriately rigorous statistical threshold and indicate this in the methods section

We addressed multiple testing using prespecified correction procedures according to the analytic context. For analyses involving individual sleep components and plasma-wide protein associations, Bonferroni correction was applied to control for multiple comparisons. Specifically, the association between the sleep health score and 2,911 plasma proteins was assessed using multivariable linear regression, and statistical significance was defined using Bonferroni correction. For subgroup and interaction analyses, functional enrichment analyses, and mediation analyses, false discovery rate correction was applied. All statistical tests were two-sided, and statistical significance was defined as Bonferroni- or FDR-adjusted $P < 0.05$. These thresholds and correction procedures are explicitly described in the Statistical analysis section of the manuscript.

5. Please declare to what extent have AI chatbots been used in developing your paper and to which parts of the paper did they contribute	Artificial intelligence chatbots were used only to assist with language polishing, grammar correction, and improving the clarity and readability of the manuscript and response letter. We declare that AI tools were not used for data analysis, statistical processing, image generation, or for the formulation of scientific hypotheses or conclusions. The authors have carefully reviewed the final manuscript and take full responsibility for its content and scientific integrity.

Supplementary Table 2. Definition, conceptual mapping, and scoring of sleep health behaviors.

Sleep behaviors	Conceptual sleep health domain	Rationale for inclusion	0 point	1 point	Data-field [a]	Original response categorization [b]
Sleep duration	Duration	Captures habitual sleep amount; both short and long sleep duration have been associated with adverse cardiometabolic outcomes.	<7 h per day or >8 h per day	7–8 h per day	1160	<7 hours (short) versus 7-8 hours (medium) versus >8 hours (long)
Non-restorative sleep	Satisfaction/restoration	Reflects subjective restoration and difficulty waking, related to perceived sleep quality.	"Not at all easy", "Not very easy"	"Fairly easy", "Very easy"	1170	"Not at all easy", "Not very easy" versus "Fairly easy", "Very easy"
Chronotype	Timing/circadian preference	Reflects circadian preference and sleep timing tendency.	Evening or morning type	Intermediate chronotype (more evening than morning or more morning than evening)	1180	"Definitely a 'morning' person" (morning chronotype) versus "More a 'morning' than 'evening' person", "More an 'evening' than 'morning' person" (intermediate chronotype) versus "Definitely an 'evening' person (evening chronotype)
Napping	Daytime sleep behavior	Reflects daytime sleep behavior and potential compensation for nocturnal sleep problems or daytime sleepiness.	Usually	Never/rarely or sometimes	1190	"Usually" versus "Sometimes", "Never/rarely"
Insomnia	Sleep continuity	Reflects difficulty initiating or maintaining sleep and nocturnal sleep disruption.	Usually	Never/rarely or sometimes	1200	"Usually" versus "Sometimes", "Never/rarely"
Snoring	Sleep-disordered breathing symptom	Serves as a self-reported marker of obstructive sleep-related symptoms.	Yes	No	1210	"Yes" versus "No"
Daytime dozing	Alertness/daytime impairment	Captures unintended daytime sleepiness and impaired wakefulness.	Often or all of the time	Never/rarely or sometimes	1220	"Often", "All of the time " versus "Sometimes", "Never/rarely"

[a] All the data-field can be accessed through the web of UK Biobank (https://biobank.ndph.ox.ac.uk/showcase/search.cgi)

[b] Participants who selected responses such as “prefer not to answer”, “do not know” or who skipped the question were classified as having missing data for that item and were excluded from the analysis.

Supplementary Table 3. Definitions of outcome, including cardiovascular disease and overall mortality.

Outcomes	ICD-10 codes	Data-field [a]
Cardiovascular disease		
Coronary heart disease	I20-I25	131296~131307
Stroke	I60-I64	131360~131369
Atrial fibrillation	I48	131350~131351
Heart failure	I50	131354~131355
Cardiovascular disease mortality	I00-I99	40000, 40001, 40007
Overall mortality	-	40000, 40001, 40007

Abbreviation: ICD-10: International Classification of Diseases, tenth version.

[a] All the data-field can be accessed through the web of UK Biobank (https://biobank.ndph.ox.ac.uk/showcase/search.cgi)

Supplementary Table 4. Definitions of covariates.

Factor	Data-field [a]	Description
Age	21022	Age at recruitment.
Sex	31	Male and female.
Ethnicity	21000	White and non-White.
Townsend deprivation index	22189	Townsend deprivation index at recruitment.
Employment	6142	Employed, retired, or unemployed/unable to work.
Body mass index	21001	Body mass index
Education	6138	High, moderate, low, or other.
Smoking	20116	Smoking status: never, previous, or current.

Alcohol	20117	Alcohol drinker status: never, previous, or current.
Physical activity	22034	Summed minutes activity.
Family history of heart disease and stroke	20107, 20110, 20111	Family history was collected through screening questions, for example: “Has your father, mother, or any sibling ever been diagnosed with stroke, or heart disease?” Responses were recorded separately for parents and siblings and combined into a summary variable.

[a] All the data-field can be accessed through the web of UK Biobank (https://biobank.ndph.ox.ac.uk/showcase/search.cgi)

Supplementary Table 5. Baseline characteristics of included and excluded participants.

	**Included cohort**	**Excluded cohort**	**Missing in excluded**	**SMD**
Number of participants	257,509	244,427		
Age, years	57.00 (49.00, 62.00)	59.00 (51.00, 64.00)	0 (0.0)	0.195
Female (%)	138261 (53.7)	134775 (55.1)	0 (0.0)	0.029
White (%)	245660 (95.4)	226513 (93.7)	2774 (1.1)	0.073
Employment (%)			5739 (2.3)	0.182
Employed	167497 (65.0)	134277 (56.3)		
Retired	74017 (28.7)	84154 (35.3)		
Unemployed/unable to work	15995 (6.2)	20257 (8.5)		
Education (%)			10119 (4.1)	0.249
High	135333 (52.6)	101741 (43.4)		
Moderate	46025 (17.9)	40298 (17.2)		
Low	42292 (16.4)	40908 (17.5)		
Other	33859 (13.1)	51361 (21.9)		
Townsend deprivation index	-2.31 (-3.73, 0.14)	-1.92 (-3.53, 0.99)	621 (0.3)	0.152

Body mass index, kg/m2	26.52 (23.98, 29.57)	27.00 (24.32, 30.27)	3103 (1.3)	0.114
Physical activity, min/week	100.00 (60.00, 180.00)	100.00 (50.00, 180.00)	117100 (47.9)	0.017
Smoking (%)			2948 (1.2)	0.061
Never	142125 (55.2)	131095 (54.3)		
Current	24984 (9.7)	27945 (11.6)		
Former	90400 (35.1)	82439 (34.1)		
Alcohol (%)			1652 (0.7)	0.098
Never	9407 (3.7)	12945 (5.3)		
Current	239992 (93.2)	219860 (90.6)		
Former	8110 (3.1)	9970 (4.1)		
Family history of heart disease and stroke (%)	150066 (58.3)	132601 (54.2)	0 (0.0)	0.081
Sleep duration (%)			4210 (1.7)	0.093
Short (< 7 h)	59618 (23.2)	63493 (26.4)		
Medium (7-8 h)	179424 (69.7)	156879 (65.3)		
Long (> 8 h)	18467 (7.2)	19845 (8.3)		
Non-restorative sleep (%)	45146 (17.5)	44427 (18.6)	5666 (2.3)	0.028
Chronotype (%)			58257 (23.8)	0.03
Morning	68736 (26.7)	51475 (27.6)		
Intermediate	166041 (64.5)	117391 (63.1)		
Evening	22732 (8.8)	17304 (9.3)		
Napping (%)	12709 (4.9)	14166 (5.8)	1892 (0.8)	0.04
Insomnia (%)	69115 (26.8)	72122 (29.7)	1503 (0.6)	0.063
Snoring (%)	94780 (36.8)	78428 (37.8)	37007 (15.1)	0.021
Daytime dozing (%)	6470 (2.5)	7615 (3.2)	3733 (1.5)	0.039

Values are given as median (interquartile range), or n (%). Standardized mean differences (SMDs) were calculated to assess the magnitude of differences between

groups, with values <0.1 generally indicating negligible imbalance. SMD, standardized mean difference.

Supplementary Table 6. Associations of the SHS with cardiovascular events and mortality stratified by sex.

Outcome	Male		P_{FDR}	Female		P_{FDR}	P interaction (FDR)
	Case/Total	HR (95% CI)		Case/Total	HR (95% CI)		
**Coronary heart disease**							
Per 1-point increase in SHS	12187/110562	0.93 (0.92, 0.95)	<0.001	6927/134671	0.90 (0.89, 0.92)	<0.001	0.167
Sleep health score							0.641
6-7 (best sleep)	5644/57169	1.00 (ref)		3251/74366	1.00 (ref)		
5	3568/31608	1.08 (1.03, 1.12)	0.003	1901/35221	1.10 (1.04, 1.17)	0.004	
3-4	2727/20408	1.21 (1.15, 1.27)	<0.001	1632/23830	1.27 (1.20, 1.35)	<0.001	
0-2 (worst sleep)	248/1377	1.36 (1.19, 1.54)	<0.001	143/1254	1.79 (1.51, 2.12)	<0.001	
**Stroke**							
Per 1-point increase in SHS	3917/117123	0.97 (0.94, 1.00)	0.025	2717/136826	0.94 (0.91, 0.98)	<0.001	0.378
Sleep health score							0.193
6-7 (best sleep)	1859/59947	1.00 (ref)		1348/75225	1.00 (ref)		
5	1135/33523	1.02 (0.95, 1.10)	0.544	729/35828	1.04 (0.95, 1.13)	0.486	
3-4	852/22112	1.11 (1.02, 1.20)	0.019	586/24446	1.15 (1.04, 1.27)	0.008	
0-2 (worst sleep)	71/1541	1.10 (0.86, 1.39)	0.457	54/1327	1.72 (1.30, 2.26)	<0.001	
**Atrial fibrillation**							
Per 1-point increase in SHS	10353/116387	0.97 (0.95, 0.99)	<0.001	6054/137154	0.97 (0.94, 0.99)	0.002	0.752
Sleep health score							0.701
6-7 (best sleep)	4867/59503	1.00 (ref)		3052/75316	1.00 (ref)		
5	3034/33282	0.97 (0.91, 1.03)	0.486	1589/35935	1.04 (0.99, 1.09)	0.219	
3-4	2249/22051	1.09 (1.02, 1.17)	0.011	1303/24551	1.09 (1.04, 1.15)	<0.001	

0-2 (worst sleep)	203/1551	1.39 (1.14, 1.68)	0.001	110/1352	1.15 (1.00, 1.33)	0.073	
**Heart failure**							
Per 1-point increase in SHS	5326/118334	0.92 (0.90, 0.94)	<0.001	2955/137975	0.89 (0.86, 0.92)	<0.001	0.093
Sleep health score							0.195
6-7 (best sleep)	2277/60440	1.00 (ref)		1310/75735	1.00 (ref)		
5	1569/33849	1.05 (0.96, 1.15)	0.481	785/36151	1.10 (1.03, 1.18)	0.008	
3-4	1318/22457	1.32 (1.20, 1.44)	<0.001	773/24728	1.23 (1.15, 1.32)	<0.001	
0-2 (worst sleep)	162/1588	1.90 (1.52, 2.37)	<0.001	87/1361	1.50 (1.28, 1.77)	<0.001	
**CVD mortality**							
Per 1-point increase in SHS	3391/119248	0.97 (0.94, 1.00)	0.039	1434/138261	0.95 (0.91, 1.00)	0.037	0.791
Sleep health score							0.268
6-7 (best sleep)	1525/60801	1.00 (ref)		692/75836	1.00 (ref)		
5	986/34121	0.96 (0.84, 1.09)	0.486	367/36242	1.03 (0.95, 1.12)	0.544	
3-4	793/22702	1.13 (0.99, 1.29)	0.067	338/24807	1.09 (1.00, 1.19)	0.052	
0-2 (worst sleep)	87/1624	1.69 (1.21, 2.37)	0.002	37/1376	1.14 (0.91, 1.42)	0.302	
**Overall mortality**							
Per 1-point increase in SHS	14347/119248	0.96 (0.95, 0.98)	<0.001	9568/138261	0.94 (0.92, 0.96)	<0.001	0.379
Sleep health score							0.73
6-7 (best sleep)	6652/60801	1.00 (ref)		4659/75836	1.00 (ref)		
5	4101/34121	1.05 (1.00, 1.10)	0.113	2604/36242	1.01 (0.98, 1.05)	0.544	
3-4	3232/22702	1.16 (1.10, 1.22)	<0.001	2125/24807	1.11 (1.06, 1.16)	<0.001	
0-2 (worst sleep)	362/1624	1.51 (1.30, 1.76)	<0.001	180/1376	1.30 (1.17, 1.45)	<0.001	

Multiple Cox proportional hazards regression model was adjusted for age, sex, ethnicity, Townsend deprivation index, body mass index, employment status, educational level, smoking status, alcohol consumption, physical activity, and family history of heart disease and stroke. *P* values were based on two-sided tests and were adjusted using the FDR method. HR, hazard ratio; CI, confidence interval; FDR, false discovery rate; SHS, sleep health score; CVD, cardiovascular disease.

Supplementary Table 7. Associations of the SHS with cardiovascular events and mortality stratified by age.

Outcome	Age > 60		P_{FDR}	Age ≤ 60		P_{FDR}	P interaction (FDR)
	Case/Total	HR (95% CI)		Case/Total	HR (95% CI)		
**Coronary heart disease**							
Per 1-point increase in SHS	10112/80426	0.93 (0.92, 0.95)	<0.001	9002/164807	0.90 (0.89, 0.92)	<0.001	0.216
Sleep health score							0.434
6-7 (best sleep)	4840/42578	1.00 (ref)		4055/88957	1.00 (ref)		
5	2921/22425	1.09 (1.04, 1.14)	<0.001	2548/44404	1.10 (1.05, 1.16)	<0.001	
3-4	2166/14527	1.19 (1.13, 1.25)	<0.001	2193/29711	1.31 (1.24, 1.38)	<0.001	
0-2 (worst sleep)	185/896	1.41 (1.22, 1.64)	<0.001	206/1735	1.55 (1.34, 1.78)	<0.001	
**Stroke**							
Per 1-point increase in SHS	3975/86427	0.96 (0.93, 0.98)	0.001	2659/167522	0.95 (0.92, 0.99)	0.005	0.239
Sleep health score							0.322
6-7 (best sleep)	1938/45204	1.00 (ref)		1269/89968	1.00 (ref)		
5	1105/24183	1.02 (0.95, 1.10)	0.687	759/45168	1.07 (0.98, 1.17)	0.16	
3-4	865/16004	1.17 (1.08, 1.27)	<0.001	573/30554	1.09 (0.99, 1.21)	0.111	
0-2 (worst sleep)	67/1036	1.24 (0.97, 1.58)	0.112	58/1832	1.38 (1.06, 1.81)	0.030	
**Atrial fibrillation**							
Per 1-point increase in SHS	10562/85817	0.97 (0.95, 0.98)	<0.001	5845/167724	0.96 (0.94, 0.98)	<0.001	0.327
Sleep health score							0.171
6-7 (best sleep)	5166/44806	1.00 (ref)		2753/90013	1.00 (ref)		
5	2963/24031	1.01 (0.96, 1.06)	0.687	1660/45186	1.06 (0.99, 1.12)	0.103	
3-4	2240/15934	1.10 (1.04, 1.16)	0.001	1312/30668	1.11 (1.04, 1.19)	0.005	
0-2 (worst sleep)	193/1046	1.24 (1.07, 1.43)	0.007	120/1857	1.22 (1.01, 1.47)	0.057	
**Heart failure**							
Per 1-point increase in SHS	5561/87709	0.91 (0.89, 0.93)	<0.001	2720/168600	0.90 (0.88, 0.93)	<0.001	0.161

Sleep health score							0.656
6-7 (best sleep)	2457/45764	1.00 (ref)		1130/90411	1.00 (ref)		
5	1587/24536	1.09 (1.02, 1.16)	0.01	767/45464	1.12 (1.02, 1.23)	0.03	
3-4	1358/16329	1.26 (1.17, 1.34)	<0.001	733/30856	1.30 (1.18, 1.43)	<0.001	
0-2 (worst sleep)	159/1080	1.71 (1.45, 2.01)	<0.001	90/1869	1.46 (1.17, 1.83)	0.002	
**CVD mortality**							
Per 1-point increase in SHS	3241/88473	0.95 (0.93, 0.98)	0.002	1584/169036	0.98 (0.94, 1.02)	0.426	0.891
Sleep health score							0.831
6-7 (best sleep)	1504/46063	1.00 (ref)		713/90574	1.00 (ref)		
5	907/24778	1.02 (0.94, 1.11)	0.687	446/45585	1.02 (0.90, 1.15)	0.788	
3-4	753/16521	1.14 (1.05, 1.25)	0.006	378/30988	1.05 (0.92, 1.19)	0.509	
0-2 (worst sleep)	77/1111	1.30 (1.03, 1.64)	0.038	47/1889	1.16 (0.85, 1.57)	0.394	
**Overall mortality**							
Per 1-point increase in SHS	15582/88473	0.95 (0.94, 0.96)	<0.001	8333/169036	0.95 (0.93, 0.96)	<0.001	0.064
Sleep health score							0.171
6-7 (best sleep)	7494/46063	1.00 (ref)		3817/90574	1.00 (ref)		
5	4363/24778	1.02 (0.98, 1.06)	0.316	2342/45585	1.08 (1.02, 1.14)	0.008	
3-4	3383/16521	1.13 (1.08, 1.17)	<0.001	1974/30988	1.16 (1.10, 1.23)	<0.001	
0-2 (worst sleep)	342/1111	1.47 (1.32, 1.64)	<0.001	200/1889	1.26 (1.09, 1.45)	0.005	

Multiple Cox proportional hazards regression model was adjusted for age, sex, ethnicity, Townsend deprivation index, body mass index, employment status, educational level, smoking status, alcohol consumption, physical activity, and family history of heart disease and stroke. *P* values were based on two-sided tests and were adjusted using the FDR method. HR, hazard ratio; CI, confidence interval; FDR, false discovery rate; SHS, sleep health score; CVD, cardiovascular disease.

Supplementary Table 8. Sensitivity analyses of associations between the SHS and cardiovascular events and mortality using competing risk model.

Outcome	Events/person-years	HR (95% CI)	P_{FDR}
**Coronary heart disease**			
Per 1-point increase in SHS	19,114/3,874,859	0.92 (0.91, 0.93)	<0.001
Sleep health score			
6-7 (best sleep)	8,895/2,092,031	1.00 (ref)	
5	5,469/1,053,354	1.09 (1.05, 1.13)	<0.001
3-4	4,359/689,886	1.24 (1.19, 1.28)	<0.001
0-2 (worst sleep)	391/39,587	1.48 (1.34, 1.65)	<0.001
Sleep Health Patterns			
Obstructive Symptom group	7,346/1,262,120	1.14 (1.10, 1.19)	<0.001
Mixed Sleep Disturbance group	4,482/794,135	1.27 (1.21, 1.32)	<0.001
Morning Chronotype group	2,766/618,980	1.07 (1.02, 1.12)	0.010
Non-Restorative Sleep group	1,170/343,427	1.18 (1.10, 1.26)	<0.001
Reference	3,350/856,198	1.00 (ref)	
**Stroke**			
Per 1-point increase in SHS	6,634/4,134,978	0.96 (0.94, 0.98)	<0.001
Sleep health score			
6-7 (best sleep)	3,207/2,204,112	1.00 (ref)	
5	1,864/1,128,880	1.03 (0.97, 1.09)	0.33
3-4	1,438/755,792	1.13 (1.06, 1.20)	<0.001
0-2 (worst sleep)	125/46,194	1.30 (1.08, 1.56)	0.005
Sleep Health Patterns			
Obstructive Symptom group	2,387/1,363,503	1.01 (0.94, 1.08)	0.742
Mixed Sleep Disturbance group	1,516/861,390	1.06 (0.99, 1.15)	0.099
Morning Chronotype group	983/654,753	0.95 (0.85, 1.04)	0.253
Non-Restorative Sleep group	433/357,234	1.12 (1.00, 1.25)	0.043
Reference	1,315/898,098	1.00 (ref)	
**Atrial fibrillation**			
Per 1-point increase in SHS	16,407/4,045,343	0.97 (0.96, 0.98)	<0.001
Sleep health score			
6-7 (best sleep)	7,919/2,158,853	1.00 (ref)	
5	4,623/1,103,240	1.01 (0.98, 1.05)	0.461
3-4	3,552/738,241	1.09 (1.05, 1.14)	<0.001
0-2 (worst sleep)	313/45,009	1.22 (1.09, 1.37)	<0.001
Sleep Health Patterns			
Obstructive Symptom group	6,058/1,328,149	0.99 (0.95, 1.03)	0.654
Mixed Sleep Disturbance group	3,759/841,308	1.08 (1.03, 1.13)	0.001
Morning Chronotype group	2,504/642,249	1.01 (0.96, 1.06)	0.791
Non-Restorative Sleep group	913/353,939	1.06 (0.99, 1.14)	0.121
Reference	3,173/879,699	1.00 (ref)	
**Heart failure**			

Per 1-point increase in SHS	8,281/4,165,431	0.91 (0.89, 0.93)	<0.001
Sleep health score			
6-7 (best sleep)	3,587/2,219,979	1.00 (ref)	
5	2,354/1,136,954	1.09 (1.03, 1.15)	0.002
3-4	2,091/761,982	1.26 (1.19, 1.33)	<0.001
0-2 (worst sleep)	249/46,516	1.61 (1.40, 1.84)	<0.001
Sleep Health Patterns			
Obstructive Symptom group	3,016/1,373,794	1.05 (0.98, 1.12)	0.161
Mixed Sleep Disturbance group	2,137/866,632	1.28 (1.20, 1.37)	<0.001
Morning Chronotype group	1,224/660,258	1.07 (0.99, 1.15)	0.095
Non-Restorative Sleep group	504/359,938	1.29 (1.16, 1.43)	<0.001
Reference	1,400/904,810	1.00 (ref)	
**CVD mortality**			
Per 1-point increase in SHS	4,825/4,216,460	0.97 (0.94, 0.99)	0.005
Sleep health score			
6-7 (best sleep)	2,217/2,240,156	1.00 (ref)	
5	1,353/1,151,987	1.01 (0.94, 1.08)	0.811
3-4	1,131/775,797	1.11 (1.03, 1.19)	0.008
0-2 (worst sleep)	124/48,519	1.26 (1.04, 1.52)	0.016
Sleep Health Patterns			
Obstructive Symptom group	1,767/1,391,678	0.96 (0.88, 1.04)	0.278
Mixed Sleep Disturbance group	1,122/880,952	1.05 (0.96, 1.15)	0.264
Morning Chronotype group	731/667,933	0.99 (0.90, 1.10)	0.899
Non-Restorative Sleep group	302/363,200	1.21 (1.06, 1.39)	0.004
Reference	903/912,697	1.00 (ref)	

Multiple Cox proportional hazards regression model was adjusted for age, sex, ethnicity, Townsend deprivation index, body mass index, employment status, educational level, smoking status, alcohol consumption, physical activity, and family history of heart disease and stroke. *P* values were based on two-sided tests and were adjusted using the FDR method. HR, hazard ratio; CI, confidence interval; FDR, false discovery rate; SHS, sleep health score; CVD, cardiovascular disease.

Supplementary Table 9. Sensitivity analyses of associations between the SHS and cardiovascular events and mortality (excluding events occurring within the first year of follow-up).

Outcome	Case/Total	HR (95% CI)	P_{FDR}
**Coronary heart disease**			
Per 1-point increase in SHS	18246/244365	0.92 (0.91, 0.93)	<0.001
Sleep health score			
6-7 (best sleep)	8526/131166	1.00 (ref)	
5	5202/66562	1.08 (1.04, 1.12)	<0.001
3-4	4149/44028	1.23 (1.18, 1.29)	<0.001
0-2 (worst sleep)	369/2609	1.47 (1.32, 1.63)	<0.001
Sleep Health Patterns			

Obstructive Symptom group	7000/80155	1.14 (1.09, 1.19)	<0.001
Mixed Sleep Disturbance group	4278/50417	1.26 (1.21, 1.32)	<0.001
Morning Chronotype group	2647/38892	1.07 (1.01, 1.12)	0.028
Non-Restorative Sleep group	1117/21387	1.17 (1.09, 1.26)	<0.001
Reference	3204/53514	1.00 (ref)	
**Stroke**			
Per 1-point increase in SHS	6409/253724	0.96 (0.94, 0.98)	<0.001
Sleep health score			
6-7 (best sleep)	3116/135081	1.00 (ref)	
5	1798/69285	1.03 (0.97, 1.09)	0.431
3-4	1376/46496	1.11 (1.04, 1.19)	0.002
0-2 (worst sleep)	119/2862	1.28 (1.06, 1.54)	0.015
Sleep Health Patterns			
Obstructive Symptom group	2308/83746	1.00 (0.94, 1.08)	0.989
Mixed Sleep Disturbance group	1455/52921	1.05 (0.97, 1.13)	0.307
Morning Chronotype group	947/40154	0.94 (0.87, 1.03)	0.273
Non-Restorative Sleep group	417/21860	1.10 (0.99, 1.23)	0.166
Reference	1282/55043	1.00 (ref)	
**Atrial fibrillation**			
Per 1-point increase in SHS	15855/252989	0.97 (0.96, 0.99)	<0.001
Sleep health score			
6-7 (best sleep)	7674/134574	1.00 (ref)	
5	4457/69051	1.01 (0.97, 1.05)	0.693
3-4	3427/46477	1.09 (1.04, 1.13)	<0.001
0-2 (worst sleep)	297/2887	1.20 (1.07, 1.35)	0.004
Sleep Health Patterns			
Obstructive Symptom group	5847/83378	0.98 (0.94, 1.03)	0.526
Mixed Sleep Disturbance group	3625/52788	1.07 (1.02, 1.13)	0.014
Morning Chronotype group	2418/40117	1.00 (0.95, 1.05)	0.989
Non-Restorative Sleep group	882/21879	1.05 (0.97, 1.13)	0.299
Reference	3083/54827	1.00 (ref)	
**Heart failure**			
Per 1-point increase in SHS	8112/256140	0.91 (0.89, 0.93)	<0.001
Sleep health score			
6-7 (best sleep)	3521/136109	1.00 (ref)	
5	2294/69940	1.08 (1.02, 1.14)	0.007
3-4	2054/47148	1.26 (1.20, 1.34)	<0.001
0-2 (worst sleep)	243/2943	1.61 (1.41, 1.84)	<0.001
Sleep Health Patterns			
Obstructive Symptom group	2959/84590	1.05 (0.98, 1.12)	0.235
Mixed Sleep Disturbance group	2096/53503	1.29 (1.20, 1.38)	<0.001
Morning Chronotype group	1198/40560	1.07 (0.99, 1.15)	0.181
Non-Restorative Sleep group	491/22047	1.28 (1.15, 1.42)	<0.001
Reference	1368/55440	1.00 (ref)	

**CVD mortality**			
Per 1-point increase in SHS	4733/257417	0.97 (0.94, 0.99)	0.006
Sleep health score			
6-7 (best sleep)	2181/136601	1.00 (ref)	
5	1325/70335	1.01 (0.94, 1.08)	0.874
3-4	1106/47484	1.10 (1.02, 1.18)	0.017
0-2 (worst sleep)	121/2997	1.25 (1.04, 1.51)	0.025
Sleep Health Patterns			
Obstructive Symptom group	1728/85007	0.95 (0.88, 1.03)	0.324
Mixed Sleep Disturbance group	1102/53848	1.05 (0.96, 1.15)	0.346
Morning Chronotype group	721/40775	1.00 (0.90, 1.10)	0.989
Non-Restorative Sleep group	294/22138	1.20 (1.05, 1.37)	0.016
Reference	888/55649	1.00 (ref)	
**Overall mortality**			
Per 1-point increase in SHS	23547/257141	0.95 (0.94, 0.96)	<0.001
Sleep health score			
6-7 (best sleep)	11140/136466	1.00 (ref)	
5	6604/70262	1.030 (1.00, 1.06)	0.073
3-4	5273/47425	1.130 (1.09, 1.17)	<0.001
0-2 (worst sleep)	530/2988	1.374 (1.26, 1.50)	<0.001
Sleep Health Patterns			
Obstructive Symptom group	8009/84928	0.94 (0.90, 0.97)	0.001
Mixed Sleep Disturbance group	5607/53777	1.08 (1.03, 1.12)	<0.001
Morning Chronotype group	3781/40731	1.02 (0.98, 1.07)	0.378
Non-Restorative Sleep group	1518/22111	1.15 (1.08, 1.22)	<0.001
Reference	4632/55594	1.00 (ref)	

Multiple Cox proportional hazards regression model was adjusted for age, sex, ethnicity, Townsend deprivation index, body mass index, employment status, educational level, smoking status, alcohol consumption, physical activity, and family history of heart disease and stroke. *P* values were based on two-sided tests and were adjusted using the FDR method. HR, hazard ratio; CI, confidence interval; FDR, false discovery rate; SHS, sleep health score; CVD, cardiovascular disease.

Supplementary Table 10. Sensitivity analyses of associations between multidimensional sleep health and cardiovascular events and mortality after multiple imputation for missing covariates.

Outcome	Events/person-years	HR (95% CI)	P_{FDR}
**Coronary heart disease**			
Per 1-point increase in SHS	32,592/6,112,492	0.92 (0.91, 0.93)	<0.001
Sleep health score			
6-7 (best sleep)	14,684/3,218,127	1.00 (ref)	
5	9,353/1,676,781	1.09 (1.06, 1.12)	<0.001
3-4	7,844/1,148,094	1.25 (1.22, 1.29)	<0.001
0-2 (worst sleep)	711/69,490	1.46 (1.36, 1.58)	<0.001

**Stroke**			
Per 1-point increase in SHS	11,493/6,561,351	0.95 (0.93, 0.96)	<0.001
Sleep health score			
6-7 (best sleep)	5,357/3,406,190	1.00 (ref)	
5	3,256/1,806,288	1.05 (1.01, 1.10)	0.021
3-4	2,644/1,266,839	1.16 (1.11, 1.22)	<0.001
0-2 (worst sleep)	236/82,034	1.33 (1.16, 1.52)	<0.001
**Atrial fibrillation**			
Per 1-point increase in SHS	28,084/6,413,531	0.96 (0.95, 0.97)	<0.001
Sleep health score			
6-7 (best sleep)	13,157/3,331,496	1.00 (ref)	
5	7,924/1,763,527	1.02 (0.99, 1.05)	0.159
3-4	6,448/1,238,163	1.11 (1.08, 1.15)	<0.001
0-2 (worst sleep)	555/80,346	1.18 (1.08, 1.28)	<0.001
**Heart failure**			
Per 1-point increase in SHS	15,142/6,610,593	0.91 (0.90, 0.93)	<0.001
Sleep health score			
6-7 (best sleep)	6,369/3,430,138	1.00 (ref)	
5	4,357/1,819,109	1.10 (1.06, 1.14)	<0.001
3-4	3,931/1,278,885	1.24 (1.19, 1.29)	<0.001
0-2 (worst sleep)	485/82,460	1.61 (1.47, 1.77)	<0.001
**CVD mortality**			
Per 1-point increase in SHS	9,003/6,702,068	0.95 (0.93, 0.96)	<0.001
Sleep health score			
6-7 (best sleep)	3,925/3,465,911	1.00 (ref)	
5	2,556/1,846,102	1.05 (0.99, 1.10)	0.086
3-4	2,259/1,303,893	1.15 (1.09, 1.22)	<0.001
0-2 (worst sleep)	263/86,162	1.33 (1.17, 1.51)	<0.001
**Overall mortality**			
Per 1-point increase in SHS	43,252/6,470,056	0.95 (0.94, 0.95)	<0.001
Sleep health score			
6-7 (best sleep)	19,830/3,359,266	1.00 (ref)	
5	12,164/1,780,906	1.04 (1.01, 1.06)	0.002
3-4	10,150/1,249,762	1.13 (1.11, 1.16)	<0.001
0-2 (worst sleep)	1,108/80,122	1.47 (1.38, 1.56)	<0.001

Multiple Cox proportional hazards regression model was adjusted for age, sex, ethnicity, Townsend deprivation index, body mass index, employment status, educational level, smoking status, alcohol consumption, physical activity, and family history of heart disease and stroke. *P* values were based on two-sided tests and were adjusted using the FDR method. HR, hazard ratio; CI, confidence interval; FDR, false discovery rate; SHS, sleep health score; CVD, cardiovascular disease.

Supplementary Table 11. Sensitivity analyses of associations between the alternative SHS and cardiovascular events and mortality after classifying sometimes responses as

unfavorable.

Outcome	Events/person-years	HR (95% CI)	P_{FDR}
**Coronary heart disease**			
Per 1-point increase in SHS	19,114/3,874,859	0.92 (0.91, 0.93)	<0.001
Sleep health score			
6-7 (best sleep)	3,075/894,681	1.00 (ref)	
5	4,664/1,099,888	1.08 (1.03, 1.13)	0.003
3-4	8,919/1,562,959	1.24 (1.19, 1.29)	<0.001
0-2 (worst sleep)	2,456/317,330	1.41 (1.34, 1.49)	<0.001
**Stroke**			
Per 1-point increase in SHS	6,634/4,134,978	0.97 (0.95, 0.99)	<0.001
Sleep health score			
6-7 (best sleep)	1,184/928,766	1.00 (ref)	
5	1,654/1,159,142	0.98 (0.91, 1.06)	0.637
3-4	3,024/1,689,862	1.06 (0.99, 1.14)	0.110
0-2 (worst sleep)	772/357,208	1.12 (1.02, 1.23)	0.026
**Atrial fibrillation**			
Per 1-point increase in SHS	16,407/4,045,343	0.97 (0.96, 0.98)	<0.001
Sleep health score			
6-7 (best sleep)	2,775/914,387	1.00 (ref)	
5	4,177/1,135,158	1.04 (0.99, 1.09)	0.184
3-4	7,463/1,648,174	1.06 (1.02, 1.11)	0.009
0-2 (worst sleep)	1,992/347,624	1.14 (1.07, 1.21)	<0.001
**Heart failure**			
Per 1-point increase in SHS	8,281/4,165,431	0.91 (0.90, 0.93)	<0.001
Sleep health score			
6-7 (best sleep)	1,107/935,808	1.00 (ref)	
5	1,883/1,166,823	1.12 (1.04, 1.21)	0.004
3-4	3,976/1,703,364	1.28 (1.20, 1.37)	<0.001
0-2 (worst sleep)	1,315/359,436	1.52 (1.40, 1.65)	<0.001
**CVD mortality**			
Per 1-point increase in SHS	4,825/4,216,460	0.95 (0.93, 0.97)	<0.001
Sleep health score			
6-7 (best sleep)	739/941,441	1.00 (ref)	
5	1,088/1,178,120	0.97 (0.88, 1.07)	0.568
3-4	2,267/1,728,083	1.09 (1.00, 1.18)	0.072
0-2 (worst sleep)	731/368,816	1.25 (1.13, 1.39)	<0.001
**Overall mortality**			
Per 1-point increase in SHS	23,915/4,086,665	0.95 (0.94, 0.96)	<0.001
Sleep health score			
6-7 (best sleep)	3,891/920,221	1.00 (ref)	
5	5,803/1,146,039	1.02 (0.98, 1.06)	0.397
3-4	11,037/1,668,598	1.11 (1.07, 1.15)	<0.001
0-2 (worst sleep)	3,184/351,808	1.26 (1.20, 1.32)	<0.001

Multiple Cox proportional hazards regression model was adjusted for age, sex, ethnicity, Townsend deprivation index, body mass index, employment status, educational level, smoking status, alcohol consumption, physical activity, and family history of heart disease and stroke. *P* values were based on two-sided tests and were adjusted using the FDR method. HR, hazard ratio; CI, confidence interval; FDR, false discovery rate; SHS, sleep health score; CVD, cardiovascular disease.

Supplementary Table 12. Sensitivity analyses of associations between the alternative SHS and cardiovascular events and mortality after recoding morning and intermediate chronotypes as favorable.

Outcome	Events/person-years	HR (95% CI)	P_{FDR}
**Coronary heart disease**			
Per 1-point increase in SHS	19,114/3,874,859	0.92 (0.91, 0.93)	<0.001
Sleep health score			
6-7 (best sleep)	4,068/1,155,786	1.00 (ref)	
5	5,380/1,161,973	1.12 (1.08, 1.17)	<0.001
3-4	8,083/1,345,556	1.25 (1.20, 1.30)	<0.001
0-2 (worst sleep)	1,583/211,543	1.40 (1.32, 1.48)	<0.001
**Stroke**			
Per 1-point increase in SHS	6,634/4,134,978	0.96 (0.95, 0.98)	<0.001
Sleep health score			
6-7 (best sleep)	1,534/1,201,676	1.00 (ref)	
5	1,880/1,231,542	1.03 (0.96, 1.10)	0.435
3-4	2,714/1,463,740	1.09 (1.02, 1.16)	0.012
0-2 (worst sleep)	506/238,020	1.17 (1.05, 1.29)	0.005
**Atrial fibrillation**			
Per 1-point increase in SHS	16,407/4,045,343	0.97 (0.96, 0.98)	<0.001
Sleep health score			
6-7 (best sleep)	3,674/1,182,922	1.00 (ref)	
5	4,686/1,204,554	1.04 (1.00, 1.09)	0.086
3-4	6,773/1,425,867	1.07 (1.03, 1.12)	0.001
0-2 (worst sleep)	1,274/232,000	1.14 (1.07, 1.22)	<0.001
**Heart failure**			
Per 1-point increase in SHS	8,281/4,165,431	0.91 (0.89, 0.92)	<0.001
Sleep health score			
6-7 (best sleep)	1,491/1,210,665	1.00 (ref)	
5	2,194/1,240,928	1.14 (1.07, 1.22)	<0.001
3-4	3,719/1,474,735	1.30 (1.22, 1.38)	<0.001
0-2 (worst sleep)	877/239,103	1.54 (1.41, 1.68)	<0.001
**CVD mortality**			
Per 1-point increase in SHS	4,825/4,216,460	0.95 (0.93, 0.97)	<0.001
Sleep health score			
6-7 (best sleep)	955/1,218,638	1.00 (ref)	
5	1,286/1,253,764	1.04 (0.96, 1.13)	0.362

3-4	2,100/1,498,352	1.13 (1.04, 1.22)	0.003
0-2 (worst sleep)	484/245,706	1.31 (1.17, 1.47)	<0.001
**Overall mortality**			
Per 1-point increase in SHS	23,915/4,086,665	0.95 (0.94, 0.96)	<0.001
Sleep health score			
6-7 (best sleep)	5,114/1,190,741	1.00 (ref)	
5	6,611/1,217,291	1.05 (1.01, 1.09)	0.010
3-4	10,084/1,444,433	1.14 (1.10, 1.18)	<0.001
0-2 (worst sleep)	2,106/234,199	1.31 (1.24, 1.38)	<0.001

Multiple Cox proportional hazards regression model was adjusted for age, sex, ethnicity, Townsend deprivation index, body mass index, employment status, educational level, smoking status, alcohol consumption, physical activity, and family history of heart disease and stroke. *P* values were based on two-sided tests and were adjusted using the FDR method. HR, hazard ratio; CI, confidence interval; FDR, false discovery rate; SHS, sleep health score; CVD, cardiovascular disease.

Supplementary Table 13. Sensitivity analyses of associations between cardiovascular events and mortality, and an SHS recalculated after excluding the chronotype component.

Outcome	Events/person-years	HR (95% CI)	P_{FDR}
**Coronary heart disease**			
Per 1-point increase in SHS	19,114/3,874,859	0.92 (0.90, 0.93)	<0.001
Sleep health score			
6 (best sleep)	782/267,978	1.00 (ref)	
5	3,497/941,032	1.14 (1.05, 1.23)	0.003
3-4	10,521/2,081,152	1.28 (1.19, 1.38)	<0.001
0-2 (worst sleep)	4,314/584,697	1.54 (1.42, 1.66)	<0.001
**Stroke**			
Per 1-point increase in SHS	6,634/4,134,978	0.96 (0.95, 0.98)	<0.001
Sleep health score			
6 (best sleep)	312/276,386	1.00 (ref)	
5	1,303/980,383	1.04 (0.92, 1.17)	0.627
3-4	3,648/2,224,992	1.08 (0.96, 1.21)	0.323
0-2 (worst sleep)	1,371/653,217	1.16 (1.02, 1.31)	0.044
**Atrial fibrillation**			
Per 1-point increase in SHS	16,407/4,045,343	0.97 (0.96, 0.98)	<0.001
Sleep health score			
6 (best sleep)	741/272,523	1.00 (ref)	
5	3,110/964,638	1.02 (0.95, 1.11)	0.627
3-4	9,012/2,173,218	1.06 (0.99, 1.15)	0.177
0-2 (worst sleep)	3,544/634,965	1.16 (1.07, 1.25)	0.001
**Heart failure**			
Per 1-point increase in SHS	8,281/4,165,431	0.91 (0.89, 0.92)	<0.001
Sleep health score			
6 (best sleep)	286/278,364	1.00 (ref)	

5	1,283/987,873	1.06 (0.94, 1.21)	0.471
3-4	4,534/2,241,580	1.26 (1.12, 1.42)	<0.001
0-2 (worst sleep)	2,178/657,615	1.50 (1.33, 1.70)	<0.001
**CVD mortality**			
Per 1-point increase in SHS	4,825/4,216,460	0.95 (0.92, 0.97)	<0.001
Sleep health score			
6 (best sleep)	204/279,763	1.00 (ref)	
5	798/995,014	0.94 (0.80, 1.09)	0.518
3-4	2,629/2,268,766	1.03 (0.89, 1.18)	0.726
0-2 (worst sleep)	1,194/672,917	1.15 (0.99, 1.34)	0.114
**Overall mortality**			
Per 1-point increase in SHS	23,915/4,086,665	0.95 (0.94, 0.96)	<0.001
Sleep health score			
6 (best sleep)	1,062/273,936	1.00 (ref)	
5	4,343/971,291	0.98 (0.92, 1.05)	0.663
3-4	13,121/2,197,271	1.06 (1.00, 1.13)	0.114
0-2 (worst sleep)	5,389/644,166	1.19 (1.11, 1.27)	<0.001

Multiple Cox proportional hazards regression model was adjusted for age, sex, ethnicity, Townsend deprivation index, body mass index, employment status, educational level, smoking status, alcohol consumption, physical activity, and family history of heart disease and stroke. *P* values were based on two-sided tests and were adjusted using the FDR method. HR, hazard ratio; CI, confidence interval; FDR, false discovery rate; SHS, sleep health score; CVD, cardiovascular disease.

Supplementary Table 14. Baseline characteristics by multidimensional sleep health patterns.

	Overall	Sleep Health Patterns					*P*-value
		**Obstructive Symptom group**	**Mixed Sleep Disturbance group**	**Morning Chronotype group**	**Non-Restorative Sleep group**	**Reference**	
Number of participants	257,509	85,046	53,868	40,785	22,146	55,664	
Age, years	57.00 (49.00 - 62.00)	57.00 (50.00 - 62.00)	58.00 (51.00 - 63.00)	57.00 (50.00 - 63.00)	51.00 (45.00 - 59.00)	56.00 (48.00 - 63.00)	<0.001
Female (%)	53.69	38.98	59.43	61.12	70.15	58.63	<0.001
White (%)	95.40	95.26	95.46	94.37	94.69	96.58	<0.001
Employment (%)							<0.001
Employed	65.05	66.54	60.49	62.64	75.58	64.74	
Retired	28.74	28.25	31.20	31.69	15.58	30.20	
Unemployed/unable to work	6.21	5.21	8.31	5.67	8.83	5.06	
Education (%)							<0.001
High	52.55	53.00	47.74	51.57	59.60	54.45	
Moderate	17.87	17.85	17.71	17.24	18.00	18.47	
Low	16.42	16.04	18.07	15.89	15.05	16.35	
Other	13.15	13.10	16.47	15.30	7.36	10.74	
Townsend deprivation index	-2.31 (-3.73 - 0.14)	-2.38 (-3.77 - -0.01)	-2.14 (-3.64 - 0.51)	-2.23 (-3.69 - 0.32)	-2.08 (-3.62 - 0.51)	-2.46 (-3.81 - -0.24)	<0.001
Body mass index, kg/m2	26.52 (23.98 - 29.57)	27.72 (25.22 - 30.79)	26.60 (23.98 - 29.82)	25.86 (23.44 - 28.72)	25.33 (22.95 - 28.33)	25.59 (23.32 - 28.31)	<0.001
Physical activity, min/week	100.00 (60.00 - 180.00)	95.00 (50.00 - 170.00)	100.00 (55.00 - 180.00)	110.00 (60.00 - 180.00)	90.00 (50.00 - 150.00)	105.00 (60.00 - 180.00)	<0.001
Smoking (%)							<0.001
Never	55.19	50.83	52.93	59.68	58.36	59.49	

Current	9.70	11.15	10.39	7.80	11.14	7.65	
Former	35.11	38.02	36.67	32.51	30.50	32.87	
Alcohol (%)							<0.001
Never	3.65	2.85	3.97	5.41	3.7	3.27	
Current	93.20	94.41	92.22	90.57	93.23	94.19	
Former	3.15	2.74	3.81	4.02	3.06	2.54	
Family history of heart disease and stroke (%)	58.28	59.32	61.03	57.47	54.77	56.01	<0.001
Sleep duration (%)							<0.001
Short (< 7 h)	23.15	14.12	73.19	13.67	11.75	0.00	
Medium (7-8 h)	69.68	77.24	22.63	80.01	80.13	91.92	
Long (> 8 h)	7.17	8.64	4.18	6.32	8.12	8.08	
Non-restorative sleep (%)	17.53	16.68	14.31	2.71	100.00	0.00	<0.001
Chronotype (%)							<0.001
Morning	26.69	25.90	17.78	91.03	0.00	0.00	
Intermediate	64.48	65.02	72.91	0.00	71.34	100.00	
Evening	8.83	9.07	9.30	8.97	28.66	0.00	
Napping (%)	4.94	5.87	5.16	5.34	3.73	3.49	<0.001
Insomnia (%)	26.84	17.06	81.33	14.50	22.06	0.00	<0.001
Snoring (%)	36.81	100.00	18.07	0.00	0.00	0.00	<0.001
Daytime dozing (%)	2.51	2.97	3.68	2.12	2.61	0.92	<0.001

Values are given as median (interquartile range), or n (%). *P*-values were based on two-sided tests.

Supplementary Table 15. Baseline characteristics of participants in mediation analysis.

	Overall	Sleep health score				*P*-value
		6-7 (best sleep)	5	3-4	0-2 (worst sleep)	
Number of participants	26,984	14,168	7,432	5,023	361	
Age, years	57.00 (49.00 - 63.00)	57.00 (49.00 - 63.00)	57.00 (50.00 - 63.00)	57.00 (50.00 - 63.00)	58.00 (51.00 - 64.00)	<0.001
Female (%)	53.22	54.88	51.28	52.08	44.04	<0.001
White (%)	94.76	95.78	93.97	93.25	91.97	<0.001
Employment (%)						<0.001
Employed	63.19	65.22	63.52	58.67	39.34	
Retired	30.10	29.63	30.17	30.74	38.23	
Unemployed/unable to work	6.72	5.15	6.31	10.59	22.44	
Education (%)						<0.001
High	52.62	55.17	51.39	47.90	43.21	
Moderate	18.02	18.29	18.06	17.32	16.34	
Low	15.84	15.34	15.74	17.36	16.62	
Other	13.52	11.19	14.81	17.42	23.82	
Townsend deprivation index	-2.24 (-3.70 - 0.33)	-2.39 (-3.77 - 0.03)	-2.23 (-3.70 - 0.38)	-1.84 (-3.53 - 1.00)	-1.17 (-3.06 - 1.90)	<0.001
Body mass index, kg/m2	26.51 (23.99 - 29.55)	26.00 (23.60 - 28.78)	26.75 (24.25 - 29.83)	27.62 (24.81 - 31.09)	29.94 (26.16 - 33.98)	<0.001
Physical activity, min/week	100.00 (60.00 - 180.00)	105.00 (60.00 - 180.00)	100.00 (55.00 - 180.00)	90.00 (45.00 - 165.00)	70.00 (20.00 - 140.00)	<0.001
Smoking (%)						<0.001
Never	54.61	57.23	53.30	49.91	44.04	
Current	9.85	8.53	9.88	12.80	19.67	
Former	35.54	34.23	36.83	37.29	36.29	
Alcohol (%)						<0.001
Never	3.91	3.39	4.12	4.84	7.20	
Current	92.71	93.72	92.80	90.36	83.66	
Former	3.38	2.89	3.08	4.80	9.14	
Family history of heart disease and stroke (%)	58.30	56.68	58.61	61.72	67.87	<0.001
Sleep duration (%)						<0.001
Short (< 7 h)	23.20	6.36	31.26	56.20	58.73	
Medium (7-8 h)	69.01	90.67	57.97	28.43	10.80	
Long (> 8 h)	7.80	2.97	10.78	15.37	30.47	
Non-restorative sleep (%)	18.16	6.55	22.07	41.11	73.68	<0.001
Chronotype (%)						<0.001
Morning	26.35	15.80	35.41	42.31	31.86	
Intermediate	64.76	81.85	54.17	35.48	19.94	
Evening	8.89	2.36	10.41	22.22	48.20	
Napping (%)	5.48	0.99	4.94	14.85	62.60	<0.001

Insomnia (%)	27.22	8.03	35.40	64.84	88.37	<0.001
Snoring (%)	36.73	21.19	48.02	60.64	81.16	<0.001
Daytime dozing (%)	2.67	0.24	1.71	7.88	45.43	<0.001

Values are given as median (interquartile range), or n (%). *P*-values were based on two-sided tests.

Supplementary Table 16. Comparison of baseline characteristics between the main cohort and proteomic subset.

	Main cohort	Proteomic subset	*P*-value	SMD
Number of participants	257,509	26,984		
Age, years	57.00 (49.00 – 62.00)	57.00 (49.00 - 63.00)	<0.001	0.029
Female (%)	138216 (53.69)	14362 (53.22)	0.143	0.009
White (%)	245660 (95.40)	25570 (94.76)	<0.001	0.03
Employment (%)			<0.001	0.039
Employed	167497 (65.05)	17050 (63.19)		
Retired	74017 (28.74)	8122 (30.10)		
Unemployed/unable to work	15995 (6.21)	1812 (6.72)		
Education (%)			0.048	0.016
High	135333 (52.55)	14198 (52.62)		
Moderate	46025 (17.87)	4863 (18.02)		
Low	42292 (16.42)	4275 (15.84)		
Other	33859 (13.15)	3648 (13.52)		
Townsend deprivation index	-2.31 (-3.73 – 0.14)	-2.24 (-3.70 - 0.33)	<0.001	0.034
Body mass index, kg/m2	26.52 (23.89 – 29.57)	26.51 (23.99 - 29.55)	0.973	0
Physical activity, min/week	100.00 (60.00 – 180.00)	100.00 (60.00 - 180.00)	0.196	0.008
Smoking (%)			0.187	0.012
Never	142125 (55.19)	14736 (54.61)		
Current	24984 (9.70)	2657 (9.85)		
Former	90400 (35.11)	9591 (35.54)		
Alcohol (%)			0.01	0.019
Never	9407 (3.65)	1056 (3.91)		
Current	239992 (93.20)	25016 (92.71)		
Former	8110 (3.15)	912 (3.38)		
Family history of heart disease and stroke (%)	150066 (58.28)	15731 (58.30)	0.946	0
Sleep duration (%)			<0.001	0.024
Short (< 7 h)	59618 (23.15)	6259 (23.20)		
Medium (7-8 h)	179424 (69.68)	18621 (69.01)		
Long (> 8 h)	18467 (7.17)	2104 (7.80)		
Non-restorative sleep (%)	45146 (17.53)	4899 (18.16)	0.011	0.016
Chronotype (%)			0.476	0.008
Morning	68736 (26.69)	7110 (26.35)		
Intermediate	166041 (64.48)	17476 (64.76)		
Evening	22732 (8.83)	2398 (8.89)		
Napping (%)	12709 (4.94)	1479 (5.48)	<0.001	0.025

Insomnia (%)	69115 (26.84)	7344 (27.22)	0.185	0.008
Snoring (%)	94780 (36.81)	9910 (36.73)	0.793	0.002
Daytime dozing (%)	6470 (2.51)	721 (2.67)	0.112	0.01

Values are given as median (interquartile range), or n (%). *P*-values were based on two-sided tests. Standardized mean differences (SMDs) were calculated to assess the magnitude of differences between groups, with values <0.1 generally indicating negligible imbalance. SMD, standardized mean difference.

Supplementary Table 17. Multivariable logistic regression assessment of proteomic data availability based on joint baseline characteristics.

Analysis	Population	Participants with proteomic data	Participants without proteomic data	Outcome	Predictors	AUC (95% CI)	Interpretation
Multivariable logistic regression	Primary analytic cohort	26,984	230,525	Proteomic data availability	Demographic, socioeconomic, lifestyle, family-history, and sleep-related variables	0.518 (0.514–0.521)	Low discrimination

The dependent variable was proteomic data availability. Predictors included age, sex, ethnicity, Townsend deprivation index, body mass index, employment status, educational level, smoking status, alcohol consumption, physical activity, family history of heart disease and stroke, and seven sleep-related variables. AUC was estimated from 10-fold cross-validated predicted probabilities. An AUC close to 0.5 indicates that the joint baseline profile had little ability to distinguish participants with proteomic data from those without proteomic data.

Supplementary Table 18. Estimated mediation proportions from exploratory protein mediation analyses of the associations between the SHS and cardiovascular events and mortality.

Proteins	Outcomes	Mediation proportions (95% CI)	P_{FDR}
ACE2	Overall mortality	28.06% (5.35%, 50.72%)	<0.001
ALDH3A1	Overall mortality	30.02% (5.85%, 52.68%)	<0.001
AMBP	Overall mortality	42.54% (13.81%, 65.19%)	<0.001
C3	Overall mortality	4.85% (0.73%, 27.50%)	0.021
CA14	Overall mortality	6.81% (1.05%, 29.46%)	0.012
CCDC80	Overall mortality	10.59% (1.69%, 33.25%)	0.021
CCL15	Overall mortality	31.19% (6.16%, 53.85%)	<0.001
CCL16	Overall mortality	10.38% (1.65%, 33.04%)	<0.001
CCL20	Overall mortality	9.00% (1.41%, 31.66%)	0.031
COLEC12	Overall mortality	15.97% (2.68%, 38.63%)	0.006
CPM	Overall mortality	28.40% (5.43%, 51.06%)	<0.001

CRHBP	Overall mortality	10.93% (1.75%, 33.59%)	<0.001
EFNA4	Overall mortality	23.82% (4.33%, 46.47%)	<0.001
FABP4	Overall mortality	31.26% (6.18%, 53.92%)	0.006
GAST	Overall mortality	15.90% (2.66%, 38.55%)	<0.001
GGT1	Overall mortality	29.71% (5.77%, 52.37%)	0.036
GUSB	Overall mortality	10.17% (1.61%, 32.83%)	<0.001
HGF	Overall mortality	49.03% (15.54%, 72.97%)	<0.001
IGF2R	Overall mortality	30.35% (5.94%, 53.01%)	0.006
IL15	Overall mortality	21.03% (3.71%, 43.69%)	<0.001
INSL5	Overall mortality	11.42% (1.83%, 34.07%)	<0.001
MRC1	Overall mortality	28.16% (6.27%, 51.02%)	<0.001
OCLN	Overall mortality	26.28% (4.91%, 48.94%)	<0.001
RNASE4	Overall mortality	11.97% (1.93%, 34.62%)	0.006
SIGLEC8	Overall mortality	10.18% (1.62%, 32.84%)	0.017
STC1	Overall mortality	29.45% (5.70%, 52.11%)	0.036
TSPAN8	Overall mortality	4.18% (0.63%, 26.84%)	0.039
CCL15	Heart failure	18.93% (3.27%, 41.58%)	<0.001
CRHBP	Heart failure	5.05% (0.76%, 27.71%)	<0.001
EFNA4	Heart failure	10.13% (1.61%, 32.79%)	0.035
FABP4	Heart failure	14.06% (2.31%, 36.71%)	0.044
GAST	Heart failure	6.42% (0.98%, 29.08%)	<0.001
HGF	Heart failure	26.80% (5.03%, 49.45%)	<0.001
IL15	Heart failure	5.37% (0.82%, 28.03%)	0.020
INSL5	Heart failure	3.35% (0.50%, 26.00%)	0.044
MRC1	Heart failure	8.92% (1.40%, 31.58%)	0.044
OCLN	Heart failure	9.43% (1.49%, 32.09%)	0.020
RNASE4	Heart failure	3.82% (0.57%, 26.48%)	0.044
ACE2	CHD	15.59% (2.60%, 38.24%)	<0.001
ALDH3A1	CHD	13.60% (2.23%, 36.26%)	0.027
AMBP	CHD	16.15% (2.71%, 38.81%)	0.008
C3	CHD	3.50% (0.52%, 26.15%)	0.005
CA14	CHD	2.58% (0.38%, 25.24%)	0.043
CCDC80	CHD	1.95% (0.29%, 24.61%)	0.039
CCL15	CHD	16.60% (2.80%, 39.25%)	<0.001
CCL16	CHD	6.71% (1.03%, 29.36%)	<0.001
CCL20	CHD	3.07% (0.46%, 25.72%)	<0.001
CD4	CHD	4.26% (0.64%, 26.92%)	0.008
COLEC12	CHD	7.42% (1.15%, 30.07%)	<0.001
CPM	CHD	19.48% (3.39%, 42.14%)	<0.001
CRHBP	CHD	8.02% (1.25%, 30.67%)	<0.001
CTSZ	CHD	4.73% (0.71%, 27.38%)	0.008
CXCL17	CHD	5.20% (0.79%, 27.86%)	0.008
EFNA4	CHD	8.33% (1.30%, 30.98%)	<0.001
FABP4	CHD	15.19% (2.53%, 37.85%)	<0.001

GAST	CHD	7.21% (1.11%, 29.86%)	<0.001
GGT1	CHD	12.93% (2.10%, 35.58%)	<0.001
GUSB	CHD	7.14% (1.10%, 29.79%)	<0.001
HAVCR1	CHD	6.36% (0.97%, 29.01%)	0.014
HGF	CHD	18.26% (3.13%, 40.91%)	<0.001
HJV	CHD	4.28% (0.64%, 26.94%)	<0.001
IGF2R	CHD	23.85% (4.34%, 46.50%)	<0.001
IL15	CHD	3.20% (0.48%, 25.85%)	0.024
INSL5	CHD	3.09% (0.46%, 25.74%)	0.005
MRC1	CHD	5.19% (0.79%, 27.84%)	0.005
OCLN	CHD	9.08% (1.42%, 31.73%)	<0.001
PRAP1	CHD	5.37% (0.82%, 28.03%)	0.017
RNASE4	CHD	6.79% (1.04%, 29.45%)	0.005
SIGLEC8	CHD	14.33% (2.37%, 36.99%)	0.005
STC1	CHD	12.88% (2.10%, 35.53%)	0.029
TNFRSF10B	CHD	5.73% (0.87%, 28.38%)	0.005
GGT1	Stroke	5.38% (0.82%, 28.03%)	0.019
HGF	Stroke	16.39% (2.76%, 39.04%)	<0.001
HJV	Stroke	3.85% (0.58%, 26.51%)	0.019
IGF2R	Stroke	25.66% (4.76%, 48.31%)	0.019
IL15	Stroke	4.66% (0.70%, 27.32%)	0.019

Mediation analyses were performed using a difference-in-coefficients approach on the log-hazard ratio scale. For each protein, the total-effect and direct-effect Cox models were fitted using the same complete-case sample. The indirect effect was calculated as the difference between the total and direct effects, and the mediation proportion was calculated as the indirect effect divided by the total effect. Confidence intervals were estimated using bootstrap resampling. Mediation analyses were adjusted for age, sex, ethnicity, Townsend deprivation index, body mass index, employment status, educational level, smoking status, alcohol consumption, physical activity, and family history of heart disease and stroke. P values were based on two-sided tests and were adjusted using the FDR method. CI, confidence interval; FDR, false discovery rate; CHD, coronary heart disease.
